# Trazodone use and risk of dementia: A population-based cohort study

**DOI:** 10.1371/journal.pmed.1002728

**Published:** 2019-02-05

**Authors:** Ruth Brauer, Wallis C. Y. Lau, Joseph F. Hayes, Kenneth K. C. Man, David P. J. Osborn, Robert Howard, Joseph Kim, Ian C. K. Wong

**Affiliations:** 1 Research Department of Practice and Policy, UCL School of Pharmacy, London, United Kingdom; 2 Centre for Safe Medication Practice and Research, Department of Pharmacology and Pharmacy, Li Ka Shing Faculty of Medicine, The University of Hong Kong, Hong Kong; 3 Division of Psychiatry, University College London, London, United Kingdom; 4 Department of Medical Informatics, Erasmus University Medical Center, Rotterdam, The Netherlands; 5 Department of Social Work and Social Administration, Faculty of Social Science, The University of Hong Kong, Hong Kong; 6 Centre of Excellence for Retrospective Studies, Real World Insights, IQVIA, London, United Kingdom; 7 Faculty of Epidemiology and Population Health, London School of Hygiene and Tropical Medicine, London, United Kingdom; 8 The University of Hong Kong, Shenzhen Hospital, Shenzhen, Guangdong, China; University of Cambridge, UNITED KINGDOM

## Abstract

**Background:**

In vitro and animal studies have suggested that trazodone, a licensed antidepressant, may protect against dementia. However, no studies have been conducted to assess the effect of trazodone on dementia in humans. This electronic health records study assessed the association between trazodone use and the risk of developing dementia in clinical practice.

**Methods and findings:**

The Health Improvement Network (THIN), an archive of anonymised medical and prescribing records from primary care practices in the United Kingdom, contains records of over 15 million patients. We assessed patients from THIN aged ≥50 years who received at least two consecutive prescriptions for an antidepressant between January 2000 and January 2017. We compared the risk of dementia among patients who were prescribed trazodone to that of patients with similar baseline characteristics prescribed other antidepressants, using a Cox regression model with 1:5 propensity score matching. Patients prescribed trazodone who met the inclusion criteria (n = 4,716; 59.2% female) were older (mean age 70.9 ± 13.1 versus 65.6 ± 11.4 years) and were more likely than those prescribed other antidepressants (n = 420,280; 59.7% female) to have cerebrovascular disease and use anxiolytic or antipsychotic drugs. After propensity score matching, 4,596 users of trazadone and 22,980 users of other antidepressants were analysed. The median time to dementia diagnosis for people prescribed trazodone was 1.8 years (interquartile range [IQR] = 0.5–5.0 years). Incidence of dementia among patients taking trazodone was higher than in matched users of other antidepressants (1.8 versus 1.1 per 100 person-years), with a hazard ratio (HR) of 1.80 (95% confidence interval [CI] 1.56–2.09; *p* < 0.001). However, our results do not suggest a causal association. When we restricted the control group to users of mirtazapine only in a sensitivity analysis, the findings were very similar to the results of the main analysis. The main limitation of our study is the possibility of indication bias, because people in the prodromal stage of dementia might be preferentially prescribed trazodone. Due to the observational nature of this study, we cannot rule out residual confounding.

**Conclusions:**

In this study of UK population-based electronic health records, we found no association between trazodone use and a reduced risk of dementia compared with other antidepressants. These results suggest that the clinical use of trazodone is not associated with a reduced risk of dementia.

## Abstract 2

Ian Wong & coworkers studied UK electronic primary care records to assess the idea from animal studies that the antidepressant trazodone might be neuroprotective in early-stage dementia

## Author summary

**Why was this study done?:**

- Dementia is a growing health concern worldwide with no disease-modifying treatment available.
- Recent in vitro and animal studies have suggested that trazodone, a licensed antidepressant, might be neuroprotective against dementia.
- No studies have assessed the effects of trazodone on dementia in humans in clinical settings.

**What did the researchers do and find?:**

- We searched primary care electronic health records from The Health Improvement Network to identify patients aged ≥50 years who received at least two consecutive prescriptions for an antidepressant between January 2000 and January 2017 in the UK.
- The risk of dementia among 4,716 users of trazodone was compared to the risk of 420,280 users of other antidepressants with similar baseline characteristics, using a Cox regression model with 1:5 propensity score matching.
- The incidence of dementia in people prescribed trazodone was higher than in matched antidepressant users (1.8 versus 1.1 per 100 person-years), with a hazard ratio (HR) of 1.80 (95% confidence interval [CI] 1.56–2.09).
- The median time to dementia diagnosis for users of trazodone was 1.8 years (interquartile range [IQR] = 0.5–5.0 years).

**What do these findings mean?:**

- In this large, UK population-based study, we found no association between trazodone use and a reduced risk of dementia compared with other antidepressants.
- Trazodone users had an early onset of dementia, which appears to contradict the suggestions from animal studies that trazodone could stop or delay the onset of dementia.
- Whilst the incidence of dementia among patients taking trazodone was higher than that in patients taking other antidepressants, the risk differences were closer to zero with increasing duration of treatment, suggesting that people in the prodromal stage of dementia might be more likely to be prescribed trazodone.

## Introduction

Dementia affects more than 47 million people worldwide [1]. Global estimates suggest that the total economic costs caused by dementia increased from US$279.6 billion in 2000 to $948 billion in 2016, with an annual growth rate of 15.94%. This included costs of informal care at $95.1 billion in 2000 and $401.9 billion in 2016, with an annual growth rate of 21.50% [2]. Dementia is characterised by a decline in cognitive functioning which impacts on activities of daily living [1]. Dementia not only affects patients but also has a significant negative effect on caregivers. Caregivers of patients with dementia are significantly more stressed than caregivers for people without dementia, and suffer more serious depressive symptoms and physical problems [3]. Effective interventions in the prevention and management of dementia are urgently needed. Alzheimer dementia (AD) and vascular dementia (VD) are the most common forms, and it can be challenging to differentiate the two clinically [4]. AD is characterised by the presence of extracellular amyloid plaques and intraneuronal neurofibrillary tangles, which are accompanied by nerve cell death and tissue loss [5]. The pathophysiological causes of AD are complex but are thought to involve overactivation of the unfolded protein response (UPR) [6]. Healthy activation of the UPR usually occurs in response to an accumulation of unfolded or misfolded proteins in the endoplasmic reticulum—e.g., in an attempt to restrict a viral infection [7].

Dysregulation of the pancreatic endoplasmic reticulum kinase branch of the UPR and its downstream target, eukaryotic initiation factor 2 (eIF2α), have been identified as potential targets in the treatment of AD [6], but no safe and effective drugs acting on this pathway exist. An attempt to identify licensed drugs with anti-eIF2α therapeutic activity that could be repurposed for use in AD suggested trazodone 2 hydrochloride, a licensed antidepressant, as a potential candidate [6]. Subsequent mice studies showed that trazodone was associated with markedly reduced neuronal loss [6]. Randomised controlled trials (RCTs) of trazodone as a sleep aid in people with mild to moderate AD showed no evidence of a positive effect on cognition, and participants experienced potentially detrimental effects on short-term memory [8, 9]. The potential for a predementia neuroprotective effect of trazodone has not been examined in humans.

Utilising the UK’s primary care electronic health records, we aimed to determine whether there is an association between trazodone use and the incidence of dementia.

## Methods

The study protocol and analysis plan was approved by the Scientific Review Committee for The Health Improvement Network (THIN) database research (Reference Number: 17THIN048, June 2017; see S1 Protocol). Further ethics approval was not required for this secondary analysis of routinely collected data.

### Data source and study design

We searched the THIN database, an archive of anonymised medical and prescribing records of primary care practices in the UK, for electronic primary care health record data for people with at least two prescriptions for an antidepressant during the study period [10]. Data from THIN are demographically representative of the UK population [11]. This study used the medical records of patients registered at 744 participating practices, comprising 13,927,536 patients meeting accepted data quality criteria [12,13] and representing over 6% of the UK population [11].

### Selection of trazodone users and the comparison group

The study population was drawn from the entire population of THIN, with follow-up time from 1 January 2000 onwards. People were selected for inclusion if they were 50 years of age or older and received two or more consecutive prescriptions for an antidepressant (Chapter 4.3 of the British National Formulary [BNF]), with the first occurrence being at least 6 months after the patient’s start of follow-up at their general practice (primary care doctor’s office) to ensure incident prescribing. This was a dynamic cohort, with follow-up ending at the earliest of the following: the date the patient left the practice, the date of death, or the date of last data collection (9 January 2017). Patients were categorised as exposed if they had received two trazodone prescriptions but no exposure to any other antidepressant agent prior to trazodone use. We matched people in the trazodone-treated group to people exposed to any other antidepressant drug currently recommended as monotherapy for depression in British prescribing guidelines (comparator group) using a matching algorithm based on propensity scores [14]. Each trazodone user was matched with up to five users of other antidepressants based on the propensity score using the greedy matching algorithm [15]. We excluded potential participants from our analysis if they had any diagnosis of dementia prior to their first prescription for an antidepressant or a record of cognitive impairment, memory symptoms, or confusion.

### Trazodone exposure

Exposure was determined from prescribing records, using drug codes for individual antidepressant agents (S1 Table). The index date for each patient was the first prescribing event that qualified them for study entry (e.g., first exposure to an antidepressant). In the primary analysis, exposure was characterised as “ever exposed” versus “never exposed” to trazodone; trazodone-exposed individuals at the start of their follow-up time were classified as ever exposed for the duration of the study, regardless of any subsequent changes in therapy.

### Dementia outcome

The primary outcome was the first recording of a diagnosis of dementia after the index date, as identified from clinical records using the Read codes in S2 Table. A secondary outcome was the median time to a diagnosis of dementia. Dementia was defined as any AD, VD, or nonspecific code. Individuals with other identifiable causes for their dementia (Parkinson disease, Huntingdon disease, Pick disease, alcohol-induced dementia, dementia in other conditions, HIV, Lewy body disease, Cruetzfeldt-Jacob disease; S2 Table) were censored at the date of the diagnosis and did not count towards the total number of outcome events.

### Propensity score matching

Since the decision to prescribe a drug is likely to be influenced by a patient’s characteristics, propensity score matching was used to reduce potential bias due to nonrandomised treatment allocation [14]. Propensity score is a measure of the probability that a patient receives a certain treatment given their observed characteristics. By matching patients with similar propensity scores, only patients with similar observed characteristics are compared, and so any observed difference in the outcome between comparison groups is less likely to be due to the underlying patient differences. Propensity scores were estimated by logistic regression with the dependent variable as the treatment of interest (trazodone) and the covariates as follows: observed patient characteristics (age, sex, general practice identifier); lifestyle variables (smoking status [non-smoker, current smoker, or ex-smoker], alcohol consumption status [non-drinker, current drinker, or ex-drinker], and body mass index [underweight, normal weight, overweight, or obese]); other risk factors for dementia (medical history of depression; anxiety; sleep disorder; substance abuse; psychotic disorder; attention deficit hyperactivity disorder; personality disorder; arrhythmia; heart failure; acute or chronic ischemic heart disease, including myocardial infarction, hypertension, cerebrovascular disease; diabetes mellitus [recorded any time using Read codes on or before the index date]); current, past, and non-use of antipsychotic agents, anti-anxiety medications, and drugs listed under BNF Chapter 2 (cardiovascular system), on the basis of use on the index date; number of general practice visits in the 12 months before the index date; and area-level social deprivation (quintile of Townsend score derived from the 2001 census data) [16]. Multiple imputation was used to replace missing smoking status, drinking status, body mass index, and Townsend score. The fully conditional specification (FCS) algorithm implemented in SAS’s Proc MI was used to create 25 imputed datasets [17]. The full analysis procedure was applied on each imputed dataset separately, and the results were combined to obtain an overall estimate. This approach accounts for the variability between imputed datasets and has been demonstrated as a robust method of applying multiple imputation techniques in propensity score modelling [18]. All covariates, including the outcome variable, were included in the imputation model to minimise bias and enhance the precision of estimates [18,19]. Patients using trazodone and patients using other antidepressants were matched in a 1:5 ratio by propensity score using a greedy matching algorithm, which has been demonstrated to perform well in both actual and simulation studies [20]. Standardised differences were used to assess the differences in patient characteristics and a value of less than 0.1 was considered negligible.

### Statistical methods

We measured a hazard ratio (HR) with a 95% confidence interval (CI) for the association between trazodone use and incident dementia using stratified Cox regression, comparing patients exposed to trazodone with matched patients exposed to other antidepressants. The logarithm of HR obtained from each imputed dataset was combined using Rubin’s rules implemented in SAS’s Proc MIANALYZE [18,21]. Sensitivity analyses were conducted using only complete cases. A two-sided *p* < 0.05 was considered statistically significant. Stata version 14 (College Station, TX) and SAS version 9.4 (Cary, NC) were used for conducting statistical analyses.

### Additional analyses

To test for the robustness of a study result, we conducted a number of sensitivity analyses. Firstly, we used a strict definition of AD in which individuals had to have received a Read code specifying AD and have at least two prescriptions for a cholinesterase inhibitor medication, to reduce the likelihood of outcome misclassification. Second, we completed an analysis censoring follow-up at the end of trazodone therapy, defined as the end of the last prescription plus 90 days. Third, we conducted an analysis stratified by length of follow-up after starting trazodone therapy (trazadone use <2 years, 2–3 years, or >3 years) to assess whether any effect of trazodone varied by duration of treatment. Fourth, we performed additional analyses that removed any events recorded within 1 month, 6 months, and 12 months after the start of follow-up, because progression to dementia is a gradual process, and diagnosis of dementia soon after starting an antidepressant treatment is unlikely to be due to the effects of the drug. Lastly, we conducted an analysis with mirtazapine as the comparison drug in an attempt to further minimise the between-group differences in prescribing choice. Mirtazapine was chosen because, like trazodone, it is a sedating antidepressant.

Additionally, we carried out the following five post hoc analyses:

1. We discounted dementia diagnoses within 2 to 10 years of the start of follow-up to further investigate the risk of dementia with prolonged follow-up.
2. We conducted subgroup analyses including only patients with a diagnosis for depression and only patients with anxiety at baseline.
3. We re-analysed excluding patients with psychotic disorders (identified by a diagnosis code for psychosis or a prescription of antipsychotic drugs at baseline). Compared to other antidepressants, trazodone is more commonly used off-label for psychotic disorders [22], so this approach aimed to further reduce the potential systematic differences between comparison groups.
4. We conducted additional analyses to restrict trazodone users to those who were continuously prescribed trazodone at doses of 194 mg or above per day, the dose that was extrapolated from the mice studies [6].
5. We completed subgroup analyses among younger patients who started treatment at 60 years old or younger, because it has been suggested that disease-modifying therapy for dementia should be administered early in the risk period so that the modification of the course of the disease is possible [23].

## Results

### Patient characteristics

**Fig 1:**
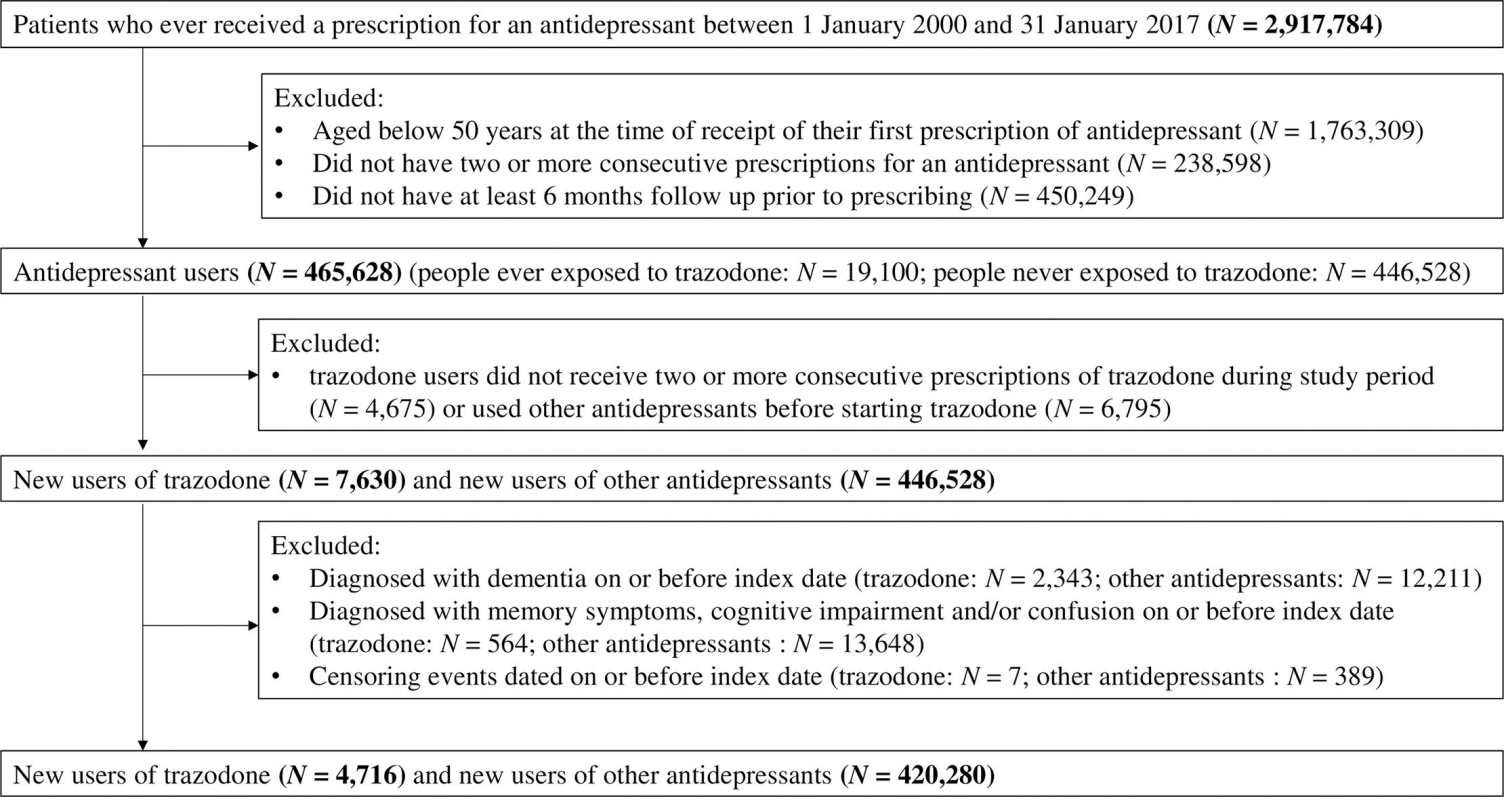
Flow of patients.

There were 465,628 patients in the THIN database had two or more consecutive prescriptions for an antidepressant and were 50 years of age or older at the time they received their first prescription on or after 1 January 2000. Of these, 4,716 first-line trazodone users met the inclusion criteria (Fig 1). We were able to match 4,596 patients who were prescribed trazadone to 22,980 users of other antidepressants. The median follow-up time of patients prescribed trazodone and those prescribed other antidepressants was 3.9 years (interquartile range [IQR] = 1.2–8.8) and 5.1 years (IQR = 2.1–9.2), respectively. A total of 18,697 out of 424,996 patients (4.4%) developed dementia during follow-up. The crude incidence rate of dementia per 100 person-years was more than twice as high in the trazodone group compared with the other antidepressant group (1.8 versus 0.7 per 100 person-years). Crude results showed that trazodone users were more likely to be diagnosed with dementia earlier (median = 1.7 years; IQR = 0.4–4.7 years) compared with users of other antidepressants (median = 4.3 years; IQR = 1.7–7.8 years). Smoking status was missing for 8.0% of all individuals who met the inclusion criteria, drinking status for 16.5%, body mass index for 15.7%, and Townsend score for 30.9%. The characteristics of patients with and without missing data are shown in S3 Table. The distributions of the observed values and the imputed values data after multiple imputation are presented in S1 Fig [19,24].

Before propensity score matching, patients prescribed trazodone were older and were more likely than those prescribed other antidepressants to have cerebrovascular disease and to use anxiolytic and/or antipsychotic drugs. After propensity score matching, all baseline characteristics were balanced between treatment groups (Table 1).

**Table 1:** Patient characteristics.

	Before PS matching		After PS matching*		Standardised difference	
	Trazodone (*N* = 4,716)	Other antidepressants (*N* = 420,280)	Trazodone (*N* = 4,596)	Other antidepressants (*N* = 22,980)	Before	After
**Age, mean ± SD**	70.9 ± 13.1	65.6 ± 11.4	70.5 ± 13.0	70.4 ± 12.0	0.43	0.01
**Sex (female)**	2,794 (59.2)	250,730 (59.7)	2,722 (59.2)	13,603 (59.2)	0.01	<0.001
**Cerebrovascular disease**	576 (12.2)	33,067 (7.9)	549 (11.9)	2,730 (11.9)	0.15	0.002
**Diabetes**	480 (10.2)	48,682 (11.6)	471 (10.2)	2,341 (10.2)	0.05	0.002
**Arrhythmias**	381 (8.1)	28,639 (6.8)	377 (8.2)	1,838 (8.0)	0.05	0.01
**Myocardial infarction**	926 (19.6)	74,790 (17.8)	910 (19.8)	4,554 (19.8)	0.05	<0.001
**Hypertension**	1,692 (35.9)	160,276 (38.1)	1,667 (36.3)	8,408 (36.6)	0.05	0.01
**Heart failure**	256 (5.4)	14,967 (3.6)	249 (5.4)	1,193 (5.2)	0.09	0.01
**Personality disorder**	50 (1.1)	2,478 (0.6)	47 (1.0)	236 (1.0)	0.05	<0.001
**ADHD**	2 (0.04)	41 (0.01)	2 (0.04)	5 (0.02)	0.02	0.01
**Psychotic disorder**	147 (3.1)	4,616 (1.1)	135 (2.9)	627 (2.7)	0.14	0.01
**Substance abuse**	226 (4.8)	12,304 (2.9)	221 (4.8)	1,071 (4.7)	0.10	0.01
**Sleep disorder**	133 (2.8)	9,044 (2.2)	127 (2.8)	657 (2.9)	0.04	0.01
**Anxiety**	856 (18.2)	74,813 (17.8)	843 (18.3)	4,204 (18.3)	0.01	0.001
**Depression**	1,403 (29.7)	149,007 (35.5)	1,389 (30.2)	6,886 (30.0)	0.12	0.01
**Smoking status**						
Current smoker	1,189 (25.2)	97,539 (23.2)	1,163 (25.3)	5,832 (25.4)	0.06	0.004
Ex-smoker	1,135 (24.1)	111,230 (26.5)	1,112 (24.2)	5,526 (24.0)	—	—
Non-smoker	2,392 (50.7)	211,511 (50.3)	2,321 (50.5)	11,622 (50.6)	—	—
**BMI, kg/m**^**2**^						
<18.5	203 (4.3)	9,081 (2.2)	186 (4.0)	888 (3.9)	0.18	0.01
18.5–24	1,827 (38.7)	147,816 (35.2)	1,775 (38.6)	8,769 (38.2)	—	—
25–29	1,757 (37.3)	157,765 (37.5)	1,715 (37.3)	8,688 (37.8)	—	—
≥30	929 (19.7)	105,618 (25.1)	920 (20.0)	4,635 (20.2)	—	—
**Drinking status**						
Current drinker	3,390 (71.9)	321,515 (76.5)	3,321 (72.3)	16,723 (72.8)	0.11	0.01
Ex-drinker	105 (2.2)	9,708 (2.3)	102 (2.2)	514 (2.2)	—	—
Non-drinker	1,221 (25.9)	89,057 (21.2)	1,173 (25.5)	5,743 (25.0)	—	—
**Antipsychotic drug use**						
Current user	429 (9.1)	10,499 (2.5)	369 (8.0)	1,789 (7.8)	0.31	0.02
Past user	114 (2.4)	3,919 (0.9)	103 (2.2)	470 (2.0)	—	—
Non-user	4,173 (88.5)	405,862 (96.6)	4,124 (89.7)	20,721 (90.2)	—	—
**Cardiovascular drug use**						
Current user	3,046 (64.6)	253,883 (60.4)	2,965 (64.5)	14,796 (64.4)	0.09	0.005
Past user	203 (4.3)	21,132 (5.0)	198 (4.3)	975 (4.2)	—	—
Non-user	1,467 (31.1)	145,265 (34.6)	1,433 (31.2)	7,209 (31.4)	—	—
**Anxiolytics drug use**						
Current user	1,557 (33.0)	97,584 (23.2)	1,501 (32.7)	7,435 (32.4)	0.22	0.01
Past user	538 (11.4)	48,869 (11.6)	516 (11.2)	2,583 (11.2)	—	—
Non-user	2,621 (55.6)	273,827 (65.2)	2,579 (56.1)	12,962 (56.4)	—	—
**Townsend score**						
1 (least deprived)	856 (18.2)	106,032 (25.2)	830 (18.1)	4,152 (18.1)	0.22	0.01
2	1,082 (22.9)	98,556 (23.5)	1,049 (22.8)	5,340 (23.2)	—	—
3	956 (20.3)	89,541 (21.3)	937 (20.4)	4,615 (20.1)	—	—
4	1,060 (22.5)	76,190 (18.1)	1,035 (22.5)	5,191 (22.6)	—	—
5 (most deprived)	762 (16.2)	49,961 (11.9)	745 (16.2)	3,682 (16.0)	—	—
**Number of general practice visits in 12 months prior to the index date, mean ± SD**	27.5 ± 20.4	28.3 ± 20.5	27.4 ± 20.4	27.4 ± 19.8	0.04	<0.001

*The propensity score analysis was performed individually for the 25 imputed datasets. The standardised difference of all covariates was less than 0.1 for all datasets after PS matching. Patient characteristics in one of the imputed datasets (of which the final result yielded the least standard error in the primary analysis) are presented for illustration.

Values are expressed as count (percentage) unless otherwise specified. Standardised difference is the absolute difference in means (for continuous variables) or proportions (for categorical variables) between trazodone users and other antidepressant users divided by the pooled SD.

**Abbreviations:** ADHD, attention deficit hyperactivity disorder; BMI, body mass index; PS, propensity score; SD, standard deviation.

### Primary analysis

A total of 445 users of trazodone (9.4%) and 18,252 users of other antidepressants (4.3%) developed dementia during follow-up. After propensity score matching, the absolute number of dementia cases was 1,997 (434 in the trazodone group and 1,563 in the other antidepressant group). The incidence of dementia in the trazodone group was higher than the incidence in the matched comparison cohort (1.8 versus 1.1 per 100 person-years) (Table 2). The HR showed an association between use of trazodone and the onset of dementia (HR = 1.80; 95% CI 1.56–2.09; *p* < 0.001) (Table 3). The median time to a diagnosis of dementia among individuals using trazodone and those taking other antidepressants was 1.8 years (IQR = 0.5–5.0 years) and 4.1 years (IQR = 1.7–7.7 years), respectively. Complete case analyses yielded similar results (S4 Table).

**Table 2:** Event rates in the primary and secondary analyses.

	Crude estimates				Adjusted estimates*			
	Trazodone		Other antidepressants		Trazodone		Other antidepressants	
Type of analysis	No. of cases/person-years	Incidence per 100 person-years	No. of cases/person-years	Incidence per 100 person-years	No. of cases/person-years	Incidence per 100 person-years	No. of cases/person-years	Incidence per 100 person-years
**Primary analysis**	445/25,142	1.8	18,252/2,516,583	0.7	434/24,793	1.8	1,563/138,953	1.1
**Secondary analyses**								
AD only	36/25,964	0.1	3,091/2,550,245	0.1	35/25,576	0.1	254/141,701	0.2
Follow-up time censored after antidepressant use	293/10,804	2.7	13,394/1,710,269	0.8	287/10,576	2.7	1,134/97,462	1.2
Trazodone versus mirtazapine	444/25,122	1.8	821/58,649	1.4	75/3,188	2.4	246/17,139	1.4
**Time-updated follow-up periods**								
Current	305/11,463	2.7	13,848/1,763,189	0.8	299/11,225	2.7	1,166/100,142	1.2
Current <2 years	210/5,788	3.6	4,709/643,224	0.7	205/5,642	3.6	401/35,241	1.1
Current 2–3 years	32/1,356	2.4	1,488/215,879	0.7	32/1,324	2.4	123/12,094	1.0
Current >3 years	63/4,319	1.5	7,651/904,086	0.8	62/4,259	1.5	642/52,807	1.2
Past	140/13,679	1.0	4,404/753,394	0.6	135/13,568	1.0	397/38,810	1.0
**Exclusion of recent dementia**								
Dementia diagnosis <31 days of follow-up	403/25,137	1.6	17,947/2,516,474	0.7	393/24,789	1.6	1,525/138,943	1.1
Dementia diagnosis <180 days of follow-up	333/25,001	1.3	16,603/2,509,140	0.7	326/24,658	1.3	1,404/138,498	1.0
Dementia diagnosis <365 days of follow-up	278/24,685	1.1	15,243/2,489,346	0.6	272/24,352	1.1	1,295/137,377	0.9

*Adjusted estimates were obtained after propensity score matching. Propensity scores were estimated by logistic regression in which the dependent variable was the treatment of interest and the covariates were the observed patient characteristics, lifestyle variables, and risk factors for dementia listed in Table 1.The propensity score analysis was performed individually for the 25 imputed datasets. The event rates were similar among the imputed datasets after propensity score matching. The event rates in one of the imputed datasets (of which the result of the primary analysis yielded the least standard error) are presented for illustration.

**Abbreviation:** AD, Alzheimer dementia.

**Table 3:** Results of the primary and secondary analyses (trazodone versus other antidepressants).

	Crude estimates		Adjusted estimates*	
Type of analysis	HR (95% CI)	*p*-Value	HR (95% CI)	*p*-Value
**Primary analysis**	2.42 (2.21–2.66)	<0.001	1.80 (1.56–2.09)	<0.001
**Secondary analyses**				
AD only	1.14 (0.82–1.58)	0.44	0.80 (0.50–1.29)	0.36
Follow-up time censored after antidepressant use	3.51 (3.13–3.94)	<0.001	2.57 (2.11–3.11)	<0.001
Trazodone versus mirtazapine	1.37 (1.22–1.54)	<0.001	1.77 (1.38–2.29)	<0.001
**Time-updated follow-up periods**				
Current	3.48 (3.11–3.90)	<0.001	2.71 (2.27–3.23)	<0.001
Current <2 years	4.88 (4.25–5.60)	<0.001	2.98 (2.43–3.65)	<0.001
Current 2–3 years	3.43 (2.42–4.87)	<0.001	2.32 (1.29–4.17)	0.005
Current >3 years	1.76 (1.38–2.26)	<0.001	1.25 (0.78–2.01)	0.35
Past	1.76 (1.48–2.08)	<0.001	1.19 (0.84–1.68)	0.33
**Exclusion of recent dementia**				
Dementia diagnosis <31 days of follow-up	2.24 (2.03–2.47)	<0.001	1.66 (1.42–1.94)	<0.001
Dementia diagnosis <180 days of follow-up	2.01 (1.81–2.24)	<0.001	1.50 (1.26–1.78)	<0.001
Dementia diagnosis <365 days of follow-up	1.83 (1.63–2.07)	<0.001	1.36 (1.12–1.65)	0.002

*Adjusted estimates were obtained after PS matching and by combining the effect estimates of the imputed datasets after PS matching, using Rubin’s rule. PSs were estimated by logistic regression in which the dependent variable was the treatment of interest and the covariates were the observed patient characteristics, lifestyle variables, and risk factors for dementia listed in Table 1.

**Abbreviations:** HR, hazard ratio; AD, Alzheimer dementia; CI, confidence interval; PS, propensity score.

### Additional analyses

When the primary outcome variable was changed from a generic dementia diagnosis to AD, no evidence of an association was found (HR = 0.80; 95% CI 0.50–1.29; *p* = 0.36). When we censored follow-up at the end of trazodone therapy, our analysis showed a stronger association between the use of trazodone and the risk of dementia compared with the results of the main analysis (HR = 2.57; 95% CI 2.11–3.11; *p* < 0.001). When the follow-up time for the trazodone-exposed group was changed to differentiate between groups with current and past trazodone exposure, a strong association was found for current trazodone exposure (HR = 2.71; 95% CI 2.27–3.23; *p* < 0.001), but we observed a weaker association with past use (HR = 1.19; 95% CI 0.84–1.68; *p* = 0.33). A stratified analysis on length of current trazodone therapy showed that short exposure to trazodone (<2 years) was more strongly associated with a higher risk of dementia (Tables 2 and 3).

When we removed any events recorded within 1 month from the start of follow-up (HR = 1.66; 95% CI 1.42–1.94; *p* < 0.001), 6 months (HR = 1.50; 95% CI 1.26–1.78; *p* < 0.001), and 12 months (HR = 1.36; 95% CI 1.12–1.65; *p* = 0.002) from the start of follow-up, the results were closer to null compared with the results of the main analysis. Results of the post hoc analysis showed that the proportion of patients with dementia in the trazodone group became similar to the proportion of patients taking other antidepressants after 3 years (Tables 4 and 5).

**Table 4:** Event rates in post hoc analyses.

	Crude estimates				Adjusted estimates*			
	Trazodone		Other antidepressants		Trazodone		Other antidepressants	
	No. of cases/person-years	Incidence per 100 person-years	No. of cases/person-years	Incidence per 100 person-years	No. of cases/person-years	Incidence per 100 person-years	No. of cases/person-years	Incidence per 100 person-years
**Patients subgroups**								
Depression	127/9,123	1.4	6,525/907,742	0.7	125/9,015	1.4	447/46,810	1.0
Anxiety	210/13,575	1.5	7,739/1,094,168	0.7	199/13,214	1.5	774/72,028	1.1
Aged 60 or below	17/10,459	0.2	1,252/1,206,576	0.1	16/10,360	0.2	66/53,093	0.1
Without psychotic disorders	371/23,175	1.6	17,133/2,425,546	0.7	370/23,025	1.6	1,263/127,723	1.0
Trazodone ≥194 mg per day	42/3,773	1.1	18,252/2,516,583	0.7	42/3,773	1.1	180/20,055	0.9
Exclusion of dementia that occurred within 1 to 10 years from index date^**^								
Excluded dementia <1 year	278/24,685	1.1	15,243/2,489,346	0.6	272/24,352	1.1	1,295/137,377	0.9
Excluded dementia <2 years	157/22,625	0.7	11,205/2,321,935	0.5	154/22,380	0.7	951/128,689	0.7
Excluded dementia <3 years	129/21,538	0.6	9,538/2,195,780	0.4	127/21,309	0.6	800/122,234	0.7
Excluded dementia <4 years	109/20,160	0.5	8,009/2,046,245	0.4	108/19,976	0.5	663/114,476	0.6
Excluded dementia <5 years	86/18,785	0.5	6,641/1,880,165	0.4	85/18,612	0.5	547/105,934	0.5
Excluded dementia <6 years	70/17,126	0.4	5,439/1,704,840	0.3	70/16,991	0.4	452/96,633	0.5
Excluded dementia <7 years	60/15,841	0.4	4,355/1,524,010	0.3	60/15,721	0.4	358/86,951	0.4
Excluded dementia <8 years	47/14,139	0.3	3,377/1,339,852	0.3	47/14,044	0.3	265/77,129	0.3
Excluded dementia <9 years	37/12,272	0.3	2,555/1,153,269	0.2	37/12,224	0.3	203/67,149	0.3
Excluded dementia <10 years	23/10,337	0.2	1,824/971,014	0.2	23/10,300	0.2	156/56,397	0.3

*Adjusted estimates were obtained after propensity score matching. Propensity scores were estimated by logistic regression in which the dependent variable was the treatment of interest and the covariates were the observed patient characteristics, lifestyle variables, and risk factors for dementia listed in Table 1. The propensity score analysis was performed individually for all the 25 imputed datasets. The event rates were similar among the imputed datasets after propensity score matching. The event rates in one of the imputed datasets (of which the result of the primary analysis yielded the least standard error) are presented for illustration.

**Exclusion of dementia that occurred within 1 to 10 years from index date for *all* patients (no subgroup analysis).

**Table 5:** Results of post hoc analyses (trazodone versus other antidepressants).

	Crude estimates		Adjusted estimates*	
	HR (95% CI)	*p*-Value	HR (95% CI)	*p*-Value
**Patients subgroups**				
Depression	1.93 (1.62–2.30)	<0.001	1.46 (1.14–1.86)	0.003
Anxiety	2.18 (1.90–2.50)	<0.001	1.58 (1.28–1.95)	<0.001
Started treatment at aged 60 or below	1.51 (0.93–2.43)	0.09	1.49 (0.67–3.29)	0.32
Without psychotic disorders	2.25 (2.03–2.49)	<0.001	1.80 (1.52–2.13)	<0.001
Trazodone ≥194 mg per day	1.52 (1.12–2.06)	0.007	1.27 (0.82–1.99)	0.28
**Exclusion of dementia that occurred within 1 to 10 years from index date**				
Excluded dementia <1 year	1.83 (1.63–2.07)	<0.001	1.36 (1.12–1.65)	0.002
Excluded dementia <2 years	1.60 (1.40–1.84)	<0.001	1.17 (0.94–1.46)	0.16
Excluded dementia <3 years	1.40 (1.20–1.64)	<0.001	0.99 (0.76–1.29)	0.95
Excluded dementia <4 years	1.34 (1.12–1.59)	0.001	0.95 (0.71–1.26)	0.70
Excluded dementia <5 years	1.33 (1.10–1.61)	0.003	0.95 (0.67–1.36)	0.78
Excluded dementia <6 years	1.25 (1.01–1.55)	0.04	0.87 (0.58–1.31)	0.50
Excluded dementia <7 years	1.23 (0.97–1.55)	0.09	0.85 (0.54–1.36)	0.50
Excluded dementia <8 years	1.30 (1.01–1.67)	0.04	0.94 (0.56–1.58)	0.81
Excluded dementia <9 years	1.30 (0.98–1.74)	0.07	0.98 (0.51–1.89)	0.96
Excluded dementia <10 years	1.35 (0.97–1.86)	0.07	1.00 (0.46–2.20)	0.99

*Adjusted estimates were obtained after propensity score matching and by combining the effect estimates of the imputed datasets after propensity score matching, using Rubin’s rule. Propensity scores were estimated by logistic regression in which the dependent variable was the treatment of interest and the covariates were the observed patient characteristics, lifestyle variables, and risk factors for dementia listed in Table 1.

**Abbreviations:** HR, hazard ratio; CI, confidence interval.

In addition, when we restricted the control group to users of mirtazapine only, the findings were very similar to the results of the main analysis (HR = 1.77; 95% CI 1.38–2.29; *p* < 0.001). Similarly, no evidence of a protective association was found in post hoc analyses when analyses were restricted to patients with depression (HR = 1.46; 95% CI 1.14–1.86; *p* = 0.003), anxiety (HR = 1.58; 95% CI 1.28–1.95; *p* < 0.001), those without psychotic disorders (HR = 1.80; 95% CI 1.52–2.13; *p* < 0.001), those who were continuously prescribed trazodone at or above 194 mg per day (HR = 1.27; 95% CI 0.82–1.99; *p* = 0.28), and those who started treatment at 60 years old or younger (HR = 1.49; 95% CI 0.67–3.29; *p* = 0.32) (Tables 4 and 5).

## Discussion

In this large, population-based study of electronic health records from the UK, we found no association between trazodone use and a reduced risk of dementia compared with other antidepressants. The results were consistent across different patient subgroups, definitions of dementia outcomes, and treatment durations as well as when comparing specific antidepressants to trazodone. The proportion of patients with dementia observed in both trazodone-treated individuals and those treated with other antidepressants were consistent with other UK population-based incidence studies [25].

We found that the incidence of dementia among patients taking trazodone was higher than that in patients taking other antidepressants. However, our results do not suggest that this association was causal—the risk differences were closer to zero with increasing duration of treatment, suggesting the possibility of reverse causality, in which people in the prodromal stage of dementia might be more likely to be prescribed trazodone. This is consistent with our observation that the median time to diagnosis of dementia among trazodone users was much shorter than that among other antidepressants users (1.8 versus 4.1 years), despite controlling for all observable prodromal characteristics using propensity scores. However, when we excluded dementia diagnoses recorded during the early years of starting treatment, the results suggested that there was no association between trazodone use and dementia diagnosed after 3 or more years after starting treatment. In particular, trazodone may be prescribed to individuals with sleep problems, which may be an independent risk factor for AD as well as an early sign of dementia [26,27]. However, the findings remained consistent when making a comparison with another sedating antidepressant (mirtazapine). Our study findings are consistent with those of the only RCT that has examined cognitive outcomes in patients with mild to moderate AD taking trazodone, which reported that trazodone had no positive effect on cognition when compared to placebo [28]. Another RCT of trazodone as a sleep aid in patients with insomnia reported small impairments in short-term memory in trazodone users [8]. Hence, neither the findings of this study nor the existing evidence supports the idea that trazodone could have a neuroprotective effect in dementia.

Because depressive symptoms can be part of the clinical presentation of unrecognised dementia, people taking an antidepressant may represent an apparently at-risk population that may go on to develop dementia [1]. The early occurrence of dementia in individuals taking trazodone shown in this study appears to contradict the idea that trazodone might stop or delay dementia onset, which had been based on indications that trazodone is neuroprotective in prion-diseased mice and tauopathy-frontotemporal dementia mice, considered to offer a plausible neurobiological model for early symptomatic dementia in humans [6]. The reason why trazodone is neuroprotective in animal models but has been ineffective in humans is unknown. However, it is not uncommon that drugs that have tested safe and effective in animal models do not translate into clinical efficacy in humans [29]. Indeed, so far, none of the drugs that slowed neurodegeneration in animal models have been successful in humans [30]. Preclinical findings that do not translate into clinical settings are particularly common in neurodegenerative disease research due to the complex mechanisms involved that are difficult to mimic in animal models [29,31]. Although animal models can replicate some aspects of the disease at a time, the progression of dementia in humans involves a spectrum of neuropathologic changes [31], which could affect the translatability of findings from animal studies to human clinical settings. A noteworthy exception is trials investigating the beneficial effects of lithium, which may improve cognitive performance in people with dementia. It is difficult to investigate the neuroprotective effects of any drug being considered for repurposing in the treatment of AD in clinical trials because of the narrow therapeutic window for CNS effects, the need to maintain physician masking to trial treatment allocation, and the long treatment period needed to assess any effect on cognitive decline. Therefore, the results of observational studies using routinely collected data are relevant. To our knowledge, this is the first clinical population-based study that has examined the risk of dementia with trazodone use. We utilised the THIN database, in which the data are representative of the UK population and reflect actual clinical practice. Data from THIN have also been used in the context of dementia in UK primary care settings to inform care [32–34].

There are some limitations to our study. All of the patients in our study received antidepressant agents. We chose not to have a nonexposed control group because we would not have been able to control for important confounders. For example, we would not be able to measure the effects of the neuropsychiatric scrutiny given to patients prescribed antidepressants. Dementia diagnoses are more likely to be made and at an earlier stage in patients who are already seeing and receiving treatment from physicians for depression or other neuropsychiatric disorders. Whilst this may have increased the likelihood of enhanced case detection in our study population, we believe that it is unlikely to have affected users of trazodone differently from those using other antidepressants. Nondifferential misclassification of undiagnosed or wrongfully diagnosed patients may have affected our results. We were also unable to assess whether the clinical diagnoses of AD met the pathological criteria for AD at autopsy. However, any bias resulting from nondifferential misclassification would be directed towards the null and is unlikely to have affected the interpretation of our results. We did not control for the adjuvant use of other antidepressants in trazodone users. So far, no other antidepressants have been suggested to affect the likelihood of developing dementia in its own right or when used concurrently with trazodone. Furthermore, given that we assessed patients taking other antidepressants as the comparator group, this would tend to have biased the results towards null and so is unlikely to affect our conclusion.

Although patients were well matched on many baseline characteristics using propensity scores, it is possible that the observed comorbidities were insufficient to identify and account for patients experiencing early symptoms of dementia. However, the early occurrence of dementia among individuals prescribed trazodone suggested a lack of association between trazodone use and delayed onset of dementia. Data in the THIN database are not collected for research purposes, and lifestyle factors such as exercise and diet are not recorded. However, these factors are not anticipated to be involved in the decision to prescribe trazodone and other antidepressants and therefore would be unlikely to introduce confounding by indication. To reduce residual confounding, we included patients who were likely to have similar indications for starting an antidepressant in the comparator group. We conducted several sensitivity analyses, and the results were found to be robust. In light of the recent literature on anticholinergic drug burden and risk for dementia, we also conducted a post hoc analysis in which we found an evenly distributed baseline exposure to anticholinergic agents (standardized differences <0.1). Furthermore, the results of a complete-case analysis controlled for the use of anticholinergic drugs in the propensity score model were consistent with the results of our original analyses. While dementia events were identified using Read codes and prescriptions, it was not possible to measure the severity of dementia at diagnosis [32]. We were also unable to obtain measurements of biomarkers for neurodegeneration among trazodone users.

Reliable continuous measures of cognitive performance to assess the extent of neurodegeneration over time are not available within the THIN dataset. However, receiving a new diagnosis for dementia is itself a clinical marker that reflects the progression of neurodegeneration. The lack of continuous measurements of cognitive function over time would only have been a problem if we had been assessing whether trazodone could slow the progression of dementia compared with other antidepressants. Because it is possible to reliably identify a diagnosis of dementia in a large clinical database such as THIN, these datasets offer an opportunity to conduct large-scale, contemporary studies with results that are generalisable to clinical practice.

## Conclusion

Using electronic health records from UK primary care, we showed that trazodone use was not associated with a reduced risk of dementia compared with other antidepressants. These results refute the suggestions from animal studies that trazodone might stop or delay the onset of dementia in patients at the prodromal stage of dementia.

## Supporting information

S1 RECORD checklist (DOCX)

S1 Protocol (DOCX)

S1 Table List of antidepressant agents. (DOCX)

S2 Table Read code list dementia. (DOCX)

S3 Table Patient characteristics. (DOCX)

S4 Table Complete case analyses. (DOCX)

S1 Fig Distributions after multiple imputation. (DOCX)

## Abbreviations

AD: Alzheimer dementia
BNF: British National Formulary
CI: confidence interval
eIF2: eukaryotic initiation factor 2
FCS: fully conditional specification
HR: hazard ratio
IQR: interquartile range
RCT: randomised controlled trial
SD: standard deviation
THIN: The Health Improvement Network
UPR: unfolded protein response
VD: vascular dementia

## References

[1] Livingston G, Sommerlad A, Orgeta V, Costafreda SG, Huntley J, Ames D, et al Dementia prevention, intervention, and care. Lancet. 2017;390(10113):2673–734. 10.1016/S0140-6736(17)31363-6 28735855

[2] Xu JF, Zhang YQ, Qiu CX, Cheng F. Global and regional economic costs of dementia: a systematic review. Lancet. 2017;390:S47–S. 10.1016/S0140-6736(17)33185-9

[3] Pinquart M, Sorensen S. Differences between caregivers and noncaregivers in psychological health and physical health: A meta-analysis. Psychol Aging. 2003;18(2):250–67. 10.1037/0882-7974.18.2.250 12825775

[4] Holmes C, Cairns N, Lantos P, Mann A. Validity of current clinical criteria for Alzheimer's disease, vascular dementia and dementia with Lewy bodies. Br J Psychiatry. 1999;174:45–50. 10211150 10.1192/bjp.174.1.45

[5] Irvine GB, El-Agnaf OM, Shankar GM, Walsh DM. Protein aggregation in the brain: the molecular basis for Alzheimer's and Parkinson's diseases. Molecular medicine. 2008;14(7–8):451–64. 10.2119/2007-00100.Irvine 18368143 PMC2274891

[6] Halliday M, Radford H, Zents KAM, Molloy C, Moreno JA, Verity NC, et al Repurposed drugs targeting eIF2&alpha;-P-mediated translational repression prevent neurodegeneration in mice. Brain. 2017;140(6):1768–83. 10.1093/brain/awx074 28430857 PMC5445255

[7] Chan SW. The unfolded protein response in virus infections. Frontiers in microbiology. 2014;5:518 10.3389/fmicb.2014.00518 25324837 PMC4179733

[8] Roth AJ, McCall WV, Liguori A. Cognitive, psychomotor and polysomnographic effects of trazodone in primary insomniacs. J Sleep Res. 2011;20(4):552–8. 10.1111/j.1365-2869.2011.00928.x 21623982 PMC3165092

[9] Camargos EF, Quintas JL, Louzada LL, Naves JO, Furioso AC, Nobrega OT. Trazodone and cognitive performance in Alzheimer disease. J Clin Psychopharmacol. 2015;35(1):88–9. 10.1097/JCP.0000000000000237 25379952

[10] Arana A, Wentworth CE, Ayuso-Mateos JL, Arellano FM. Suicide-related events in patients treated with antiepileptic drugs. New England Journal of Medicine. 2010;363(6):542–51. 10.1056/NEJMoa0909801 20818889

[11] Blak BT, Thompson M, Dattani H, Bourke A. Generalisability of The Health Improvement Network (THIN) database: demographics, chronic disease prevalence and mortality rates. Informatics in primary care. 2011;19(4):251–5. 22828580 10.14236/jhi.v19i4.820

[12] Maguire A, Blak BT, Thompson M. The importance of defining periods of complete mortality reporting for research using automated data from primary care. Pharmacoepidemiology and Drug Safety. 2009;18(1):76–83. 10.1002/pds.1688 19065600

[13] Horsfall L, Walters K, Petersen I. Identifying periods of acceptable computer usage in primary care research databases. Pharmacoepidemiology and Drug Safety. 2013;22(1):64–9. 10.1002/pds.3368 23124958

[14] Austin PC. An Introduction to Propensity Score Methods for Reducing the Effects of Confounding in Observational Studies. Multivariate Behavioral Research. 2011;46(3):399–424. 10.1080/00273171.2011.568786 21818162 PMC3144483

[15] Austin PC. A comparison of 12 algorithms for matching on the propensity score. Statistics in medicine. 2014;33(6):1057–69. 10.1002/sim.6004 24123228 PMC4285163

[16] Townsend P. Deprivation. Journal of Social Policy. 1987;16(2):125–46. Epub 2009/01/01. 10.1017/S0047279400020341

[17] van Buuren S. Multiple imputation of discrete and continuous data by fully conditional specification. Stat Methods Med Res. 2007;16(3):219–42. 10.1177/0962280206074463 17621469

[18] Leyrat C, Seaman SR, White IR, Douglas I, Smeeth L, Kim J, et al Propensity score analysis with partially observed covariates: How should multiple imputation be used? Stat Methods Med Res. 2017:962280217713032. 10.1177/0962280217713032 28573919 PMC6313366

[19] Sterne JAC, White IR, Carlin JB, Spratt M, Royston P, Kenward MG, et al Multiple imputation for missing data in epidemiological and clinical research: potential and pitfalls. BMJ. 2009;339 10.1136/bmj.b2393 19564179 PMC2714692

[20] Austin PC. Some methods of propensity-score matching had superior performance to others: results of an empirical investigation and Monte Carlo simulations. Biometrical journal Biometrische Zeitschrift. 2009;51(1):171–84. 10.1002/bimj.200810488 19197955

[21] Marshall A, Altman DG, Holder RL, Royston P. Combining estimates of interest in prognostic modelling studies after multiple imputation: current practice and guidelines. BMC Med Res Methodol. 2009;9:57 10.1186/1471-2288-9-57 19638200 PMC2727536

[22] Fagiolini A, Comandini A, Catena Dell'Osso M, Kasper S. Rediscovering trazodone for the treatment of major depressive disorder. CNS Drugs. 2012;26(12):1033–49. 10.1007/s40263-012-0010-5 23192413 PMC3693429

[23] Doody RS, Thomas RG, Farlow M, Iwatsubo T, Vellas B, Joffe S, et al Phase 3 Trials of Solanezumab for Mild-to-Moderate Alzheimer's Disease. New England Journal of Medicine. 2014;370(4):311–21. 10.1056/NEJMoa1312889 24450890

[24] Hippisley-Cox J, Coupland C. Predicting risk of osteoporotic fracture in men and women in England and Wales: prospective derivation and validation of QFractureScores. BMJ. 2009;339:b4229 10.1136/bmj.b4229 19926696 PMC2779855

[25] Matthews F, Brayne C, Medical Research Council Cognitive F, Ageing Study I. The incidence of dementia in England and Wales: findings from the five identical sites of the MRC CFA Study. Plos Medicine. 2005;2(8):e193 10.1371/journal.pmed.0020193 16111436 PMC1188245

[26] Kales HC, Gitlin LN, Lyketsos CG. Assessment and management of behavioral and psychological symptoms of dementia. British Medical Journal. 2015;350:h369 10.1136/bmj.h369 25731881 PMC4707529

[27] Ju YE, Lucey BP, Holtzman DM. Sleep and Alzheimer disease pathology—a bidirectional relationship. Nature reviews Neurology. 2014;10(2):115–9. 10.1038/nrneurol.2013.269 24366271 PMC3979317

[28] Camargos EF, Louzada LL, Quintas JL, Naves JO, Louzada FM, Nobrega OT. Trazodone improves sleep parameters in Alzheimer disease patients: a randomized, double-blind, and placebo-controlled study. Am J Geriatr Psychiatry. 2014;22(12):1565–74. 10.1016/j.jagp.2013.12.174 24495406

[29] Perrin S. Preclinical research: Make mouse studies work. Nature. 2014;507(7493):423–5. 10.1038/507423a 24678540

[30] Honig LS, Vellas B, Woodward M, Boada M, Bullock R, Borrie M, et al Trial of Solanezumab for Mild Dementia Due to Alzheimer’s Disease. New England Journal of Medicine. 2018;378(4):321–30. 10.1056/NEJMoa1705971 29365294

[31] Franco R, Cedazo-Minguez A. Successful therapies for Alzheimer's disease: why so many in animal models and none in humans? Front Pharmacol. 2014;5:146 10.3389/fphar.2014.00146 25009496 PMC4070393

[32] Rait G, Walters K, Bottomley C, Petersen I, Iliffe S, Nazareth I. Survival of people with clinical diagnosis of dementia in primary care: cohort study. British Medical Journal. 2010;341 10.1136/bmj.c3584 20688840 PMC2917003

[33] Walters K, Hardoon S, Petersen I, Iliffe S, Omar RZ, Nazareth I, et al Predicting dementia risk in primary care: development and validation of the Dementia Risk Score using routinely collected data. BMC Medicine. 2016;14 10.1186/s12916-016-0549-y 26797096 PMC4722622

[34] Grant RL, Drennan VM, Rait G, Petersen I, Iliffe S. First Diagnosis and Management of Incontinence in Older People with and without Dementia in Primary Care: A Cohort Study Using The Health Improvement Network Primary Care Database. PLoS Med. 2013;10(8). 10.1371/journal.pmed.1001505 24015113 PMC3754889

