# Supplementary material for: Trazodone use and risk of dementia: A population-based cohort study
Source: PLoS Med. 2019 Feb 5;16(2):e1002728. doi: 10.1371/journal.pmed.1002728 (PMC6363148; doi:10.1371/journal.pmed.1002728)
Supplement: S1 RECORD checklist — (DOCX) [file pmed.1002728.s001.docx]

**The RECORD statement – checklist of items, extended from the STROBE statement, that should be reported in observational studies using routinely collected health data.**

|  | **Item No.** | **STROBE items** | **Location in manuscript where items are reported** | **RECORD items** | **Location in manuscript where items are reported** |
| --- | --- | --- | --- | --- | --- |
| **Title and abstract** | | | | | |
|  | 1 | (a) Indicate the study’s design with a commonly used term in the title or the abstract (b) Provide in the abstract an informative and balanced summary of what was done and what was found | (a) **Title**: “a population-based cohort study”  (b) **Abstract**: From “In vitro…” (Line 22) until “...dementia” (Line 60) | RECORD 1.1: The type of data used should be specified in the title or abstract. When possible, the name of the databases used should be included.  RECORD 1.2: If applicable, the geographic region and timeframe within which the study took place should be reported in the title or abstract.  RECORD 1.3: If linkage between databases was conducted for the study, this should be clearly stated in the title or abstract. | 1.1.Title: “a population-based cohort study”  1.2. Abstract: “…patients in the UK” & “.. between January 2000 and January 2017…”  1.3 Not applicable: No linkage used. |
| **Introduction** | | | | | |
| Background rationale | 2 | Explain the scientific background and rationale for the investigation being reported | **Introduction**: “The authors of a recently published paper attempted to identify safe, licensed drugs with anti-eIF2α-P therapeutic activity for repurposing against AD and identified trazodone hydrochloride, a licensed antidepressant, as a potential candidate.”  &  “The potential for a pre-dementia neuroprotective effect of trazodone has not been examined in humans.” |  |  |
| Objectives | 3 | State specific objectives, including any prespecified hypotheses | **Introduction**: “Utilising the United Kingdom (UK)’s primary care electronic health records, we aimed to determine whether there is an association between trazodone use and the incidence of dementia.” |  |  |
| **Methods** | | | | | |
| Study Design | 4 | Present key elements of study design early in the paper | **Method. Data source and study design**: **“**A cohort study” |  |  |
| Setting | 5 | Describe the setting, locations, and relevant dates, including periods of recruitment, exposure, follow-up, and data collection | Method. Data source and study:  *Setting:* “primary care practices”  *Locations: “*in the UK”  **Method. Selection of trazodone users and the comparison group**:  *Relevant dates*: **“**from 1^st^ January 2000 onwards (until) the last date of data collection (9^th^ January 2017).” |  |  |
| Participants | 6 | *(a) Cohort study* - Give the eligibility criteria, and the sources and methods of selection of participants. Describe methods of follow-up  *Case-control study* - Give the eligibility criteria, and the sources and methods of case ascertainment and control selection. Give the rationale for the choice of cases and controls  *Cross-sectional study* - Give the eligibility criteria, and the sources and methods of selection of participants  *(b) Cohort study* - For matched studies, give matching criteria and number of exposed and unexposed  *Case-control study* - For matched studies, give matching criteria and the number of controls per case | *(a)* **Method. Selection of trazodone users and the comparison group**: “People were selected for inclusion if they were 50 years of age or older and received two or more consecutive prescriptions for an antidepressant, with the first occurrence being at least 6 months after the patient’s start of follow up at their general practice.”  *(b) “*We matched people in the trazodone treated group to people exposed to any other antidepressant drug (…) using a matching algorithm based on propensity scores. Each trazodone user was matched with up to 5 non-trazodone antidepressant users based on the propensity score using the greedy matching algorithm.” | RECORD 6.1: The methods of study population selection (such as codes or algorithms used to identify subjects) should be listed in detail. If this is not possible, an explanation should be provided.  RECORD 6.2: Any validation studies of the codes or algorithms used to select the population should be referenced. If validation was conducted for this study and not published elsewhere, detailed methods and results should be provided.  RECORD 6.3: If the study involved linkage of databases, consider use of a flow diagram or other graphical display to demonstrate the data linkage process, including the number of individuals with linked data at each stage. | 6.1. **Method. Selection of trazodone users and the comparison group: “**People were selected for inclusion if they were 50 years of age or older and received two or more consecutive prescriptions for an antidepressant (Chapter 4.3 of the British National Formulary [BNF]).”  6.2. Not applicable  6.3. Not applicable |
| Variables | 7 | Clearly define all outcomes, exposures, predictors, potential confounders, and effect modifiers. Give diagnostic criteria, if applicable. | **Method.**  **Trazadone Exposure & Dementia outcome & Propensity Score Matching** | RECORD 7.1: A complete list of codes and algorithms used to classify exposures, outcomes, confounders, and effect modifiers should be provided. If these cannot be reported, an explanation should be provided. | **7.1. Supporting Information file 2:** Supplemental Table 1 – List of antidepressant agents.  Supplemental Table 2 – Read code list Dementia. |
| Data sources/ measurement | 8 | For each variable of interest, give sources of data and details of methods of assessment (measurement).  Describe comparability of assessment methods if there is more than one group | **Trazadone Exposure: “**Exposure was determined by prescribing records, using drug codes…”  **Dementia outcome: “…**first recording of a diagnosis of dementia after the index date, as identified from clinical records, using the Read codes” |  |  |
| Bias | 9 | Describe any efforts to address potential sources of bias | **Propensity Score Matching**: “Propensity score matching was used to reduce potential bias due to non-randomised treatment allocation.”  **Additional Analyses:** From **“**The following sensitivity analyses…” until “…modification of the course of the disease is possible.” |  |  |
| Study size | 10 | Explain how the study size was arrived at | **Selection of trazodone users and the comparison group: “**The study population was drawn from the entire THIN population, with follow-up time from 1^st^ January 2000 onwards” |  |  |
| Quantitative variables | 11 | Explain how quantitative variables were handled in the analyses. If applicable, describe which groupings were chosen, and why | **Trazodone exposure: “**In the primary analysis, exposure was characterised as “ever exposed” versus “never exposed” to trazodone”  **Propensity Score Matching**: “…smoking status [non-, current-, ex-smoker], drinking status [non-, current-, ex-drinker], and body mass index [underweight, normal weight, overweight, obese], … medical history [recorded any time using Read codes on or before the index date]) and current, past, and non-use (based on use on the index date) of drugs as well as the number of general practice visits in the 12 months prior to the index date and area level social deprivation (quintile of Townsend score derived from the 2001 census data). |  |  |
| Statistical methods | 12 | (a) Describe all statistical methods, including those used to control for confounding  (b) Describe any methods used to examine subgroups and interactions  (c) Explain how missing data were addressed  (d) *Cohort study* - If applicable, explain how loss to follow-up was addressed  *Case-control study* - If applicable, explain how matching of cases and controls was addressed  *Cross-sectional study* - If applicable, describe analytical methods taking account of sampling strategy  (e) Describe any sensitivity analyses | (a) **Propensity Score Matching & Statistical Methods:** “….Cox regression, comparing patients exposed to trazodone with matched patients exposed to other antidepressants.  (b) **Statistical Methods: “**Sensitivity analyses were conducted using only complete cases.”  (c)**Propensity Score Matching: “**The Fully Conditional Specification (FCS) algorithm implemented in SAS’s Proc MI was used to create twenty five imputed datasets.” **Statistical Methods: “**The logarithm of HR obtained from each imputed dataset were combined using Rubin’s rules implemented in SAS’s Proc MIANALYZE.”  (d) Not applicable  (e) **Additional Analyses** |  |  |
| Data access and cleaning methods |  | .. |  | RECORD 12.1: Authors should describe the extent to which the investigators had access to the database population used to create the study population.  RECORD 12.2: Authors should provide information on the data cleaning methods used in the study. | 12.1. **Selection of trazodone users and the comparison group: “**The study population was drawn from the entire THIN population, with follow-up time from 1^st^ January 2000 onwards”  12.2. **Selection of trazodone users and the comparison group** (entire paragraph) |
| Linkage |  | .. |  | RECORD 12.3: State whether the study included person-level, institutional-level, or other data linkage across two or more databases. The methods of linkage and methods of linkage quality evaluation should be provided. | 12.3. Not applicable – No data linkage. |
| **Results** | | | | | |
| Participants | 13 | (a) Report the numbers of individuals at each stage of the study (*e.g.*, numbers potentially eligible, examined for eligibility, confirmed eligible, included in the study, completing follow-up, and analysed)  (b) Give reasons for non-participation at each stage.  (c) Consider use of a flow diagram | (a) **Figure 1 (**study flow diagram)  (b) **Figure 1**  (c) **Figure 1** | RECORD 13.1: Describe in detail the selection of the persons included in the study (*i.e.,* study population selection) including filtering based on data quality, data availability and linkage. The selection of included persons can be described in the text and/or by means of the study flow diagram. | 13.1. **Figure 1.** |
| Descriptive data | 14 | (a) Give characteristics of study participants (*e.g.*, demographic, clinical, social) and information on exposures and potential confounders  (b) Indicate the number of participants with missing data for each variable of interest  (c) *Cohort study* - summarise follow-up time (*e.g.*, average and total amount) | (a) **Table 1** & **Results-** **Patient Characteristics & Supplemental Table 3**  (b) **Supplemental Table 3 & Supplemental Figure 1**: “The distributions of the observed values and the imputed values data after multiple imputation are presented in Supplemental Figure 1”  (c) **Patient Characteristics: “**The median follow-up time of patients prescribed trazodone and those prescribed other antidepressants were 3.9 years (interquartile range [IQR]=1.2 to 8.8) and 5.1 years (IQR=2.1 to 9.2) respectively.” |  |  |
| Outcome data | 15 | *Cohort study* - Report numbers of outcome events or summary measures over time  *Case-control study* - Report numbers in each exposure category, or summary measures of exposure  *Cross-sectional study* - Report numbers of outcome events or summary measures | **Table 2 & Primary Analysis: “**A total of 18,697 out of 424,996 patients (4.4%) developed dementia during follow-up. This included 445 trazodone users (9.4%) and 18,252 other antidepressants users (4.3%).” |  |  |
| Main results | 16 | (a) Give unadjusted estimates and, if applicable, confounder-adjusted estimates and their precision (e.g., 95% confidence interval). Make clear which confounders were adjusted for and why they were included  (b) Report category boundaries when continuous variables were categorized  (c) If relevant, consider translating estimates of relative risk into absolute risk for a meaningful time period | (a) **Table 3**: Crude HR: 2.42 (2.21-2.66) &  **Primary Analysis: “…..**The HR showed an association between use of trazodone and the onset of dementia (HR=1.80, 95%CI=1.56-2.09)”  (b) **Table 1. Patient characteristics.**  **(c) Table 2. Event rates in the primary and secondary analyses: “**Incidence per 100 person-years” |  |  |
| Other analyses | 17 | Report other analyses done—e.g., analyses of subgroups and interactions, and sensitivity analyses | **Additional Analyses:** From **“**When the primary outcome….” Until “……(HR=1.49, 95%CI=0.67-3.29) (Table 4&5).” |  |  |
| **Discussion** | | | | | |
| Key results | 18 | Summarise key results with reference to study objectives | **Discussion: “**In this large UK population-based study, we found no association between trazodone use and a reduced rate of dementia when compared to other antidepressants.” |  |  |
| Limitations | 19 | Discuss limitations of the study, taking into account sources of potential bias or imprecision. Discuss both direction and magnitude of any potential bias | **Discussion** – From “There are some limitations to our study….” until “….. highly generalisable to clinical practice.” | RECORD 19.1: Discuss the implications of using data that were not created or collected to answer the specific research question(s). Include discussion of misclassification bias, unmeasured confounding, missing data, and changing eligibility over time, as they pertain to the study being reported. | **Discussion**: From Line 392 onwards “There are some limitations to our study…..”. Particularly: “THIN data are not collected for research purposes, ….” |
| Interpretation | 20 | Give a cautious overall interpretation of results considering objectives, limitations, multiplicity of analyses, results from similar studies, and other relevant evidence | **Conclusion**: “This large population-based study of the UK showed that the use of trazodone was not associated with a reduced risk of dementia when compared to other antidepressants. The results do not confirm the suggestions by animal studies. Caution is needed when speculating about the effect of trazodone on dementia in clinical practice unless any benefit of trazodone in relation to dementia has been proven in humans.” |  |  |
| Generalisability | 21 | Discuss the generalisability (external validity) of the study results | **Discussion**: “We utilised the THIN database, in which the data are generalizable to the UK population and reflect actual clinical practice.” |  |  |
| **Other Information** | | | | | |
| Funding | 22 | Give the source of funding and the role of the funders for the present study and, if applicable, for the original study on which the present article is based | **Funding:** None. |  |  |
| Accessibility of protocol, raw data, and programming code |  | .. | **Contributors:** “Study concept and design: RB, WCYL, JFH, KKCM, DPJO, ICKW; Acquisition, analysis, or interpretation of data: RB, WCYL, JFH, KKCM, DPJO, RH, JK, ICKW; Drafting of the manuscript: RB, WCYL, JFH; Critical revision of the manuscript for important intellectual content: RB, WCYL, JFH, KKCM, DPJO, RH, JK, ICKW; Statistical analysis: RB, WCYL; ” | RECORD 22.1: Authors should provide information on how to access any supplemental information such as the study protocol, raw data, or programming code. | **22.1. Supporting Information**. The study protocol and exposure &s outcome code lists are available as supplemental files. |

*Reference: Benchimol EI, Smeeth L, Guttmann A, Harron K, Moher D, Petersen I, Sørensen HT, von Elm E, Langan SM, the RECORD Working Committee. The REporting of studies Conducted using Observational Routinely-collected health Data (RECORD) Statement. *PLoS Medicine* 2015; in press.

*Checklist is protected under Creative Commons Attribution ([CC BY](http://creativecommons.org/licenses/by/4.0/)) license.
