# Supplementary material for: Trazodone use and risk of dementia: A population-based cohort study
Source: PLoS Med. 2019 Feb 5;16(2):e1002728. doi: 10.1371/journal.pmed.1002728 (PMC6363148; doi:10.1371/journal.pmed.1002728)
Supplement: S1 Protocol — (DOCX) [file pmed.1002728.s002.docx]

Title of the study

Impact of trazodone use on Dementia outcomes.

Background

*Describe the context in which the research question arises, referring to related research. Furthermore, state why the proposed research is important and who will benefit from the results*.

Alzheimer's disease (AD) is a degenerative neurological disease primarily characterised by the presence of extracellular amyloid plaques and intraneuronal neurofibrillary tangles which are believed to lead to nerve cell death and tissue loss (Irvine et al., 2008). Over time, the brain shrinks dramatically, affecting memory and cognitive skills. The pathophysiological causes of AD are complex, and are thought to involve overactivation of the unfolded protein response (UPR) (Halliday et al., 2017). Healthy activation of the UPR usually occurs in response to an accumulation of unfolded or misfolded [proteins](https://en.wikipedia.org/wiki/Protein) in the endoplasmic reticulum (ER) – for instance, in an attempt to restrict a viral infection (Chan et al., 2014). Dysregulation of the pancreatic endoplasmic reticulum kinase (PERK) branch of the UPR, and its downstream target, the phosphorylated alpha subunit of eukaryotic initiation factor 2 (eIF2α), have been identified as potentially prominent targets to treat AD (Halliday et al., 2017). However, safe and effective drugs acting on this pathway do not yet exist. Authors of a recently published paper attempted to identify safe, licensed drugs with anti-eIF2α-P therapeutic activity for repurposing and identified trazodone hydrochloride, a licensed anti-depressant, as a potential target (Halliday et al., 2017). Subsequent mice studies showed trazodone was associated with markedly reduced neuronal loss (Halliday et al., 2017). This beneficial effect of trazodone has not been demonstrated in humans.

Trazodone has been prescribed in patients with depression since the 1970s (Fagiolini et al., 2013; Linde et al., 2015) and is still widely used (with 1,083,974 items dispensed in 2015 in England; Health and Social Care Information Centre, 2016). This study, using routine primary care data, will add substantially to the evidence base regarding the effect of trazodone on dementia, with a particular emphasis on Alzheimer’s disease, in clinical practice, and will be of great value when making decisions about where to focus resources for further clinical research.

Purpose

**Aim**

To assess the association between the prescribing of trazodone on the risk of being diagnosed dementia in UK primary care.

**Objectives**

To estimate the rate ratio for dementia in people who receive treatment with antidepressants, comparing individuals treated with trazodone to people treated with agents other than trazodone.

Data source

*Explain why the data source (THIN) has been chosen, with regards to data availability and quality.*

THIN data provide us with an opportunity to study the relationship between trazodone and dementia. THIN offers an ideal source of observational data to examine whether trazodone is protective against dementia by determining the effect on dementia onset in a large population. The THIN database contains treatment, comparator and outcome data. Both prescribing and outcome data are likely to be very accurate.

Methods

*Describe the study design, e.g. case-control, cohort or cross-sectional study. Describe the procedure for selecting both cases and controls, where applicable.*

This is a hypothesis testing study using a cohort study design. The procedure for selecting both cases and controls is described in detail below.

Study design

*Describe the study design, e.g. case-control, cohort or cross-sectional study.*

We will conduct a cohort study comparing the rate of dementia diagnosis in people treated with trazodone with the rate of dementia in people being treated with non-trazodone antidepressant drugs between 2000 and 2017. Individuals exposed to trazodone will be matched to users of other antidepressant agents using propensity scores. This design will minimise confounding because both the exposed and comparator group are likely to have similar underlying health problems related to psychiatric conditions which may be associated with dementia. Due to the cohort design we will also be able to examine whether any effect of trazodone changes over time.

Study population

*Define the population on which the study will focus, with brief inclusion and exclusion criteria. Specify data quality criteria (include time frame for data selection).*

The study population will be drawn from the entire THIN population. People will be selected for inclusion if they are 50 years of age or older and have received two or more consecutive prescriptions for trazodone (BNF chapter: 4.3.1.), with the first occurrence being at least 6 months after the patient’s start of follow up at their GP practice. This is to increase the likelihood that we are studying new users of trazodone, which will be of importance when we assess any effect of treatment duration on dementia risk. Eligibility will begin in 2000 to ensure completeness and accuracy of data. The incidence of dementia increases from one in ten of those over 65 to almost half of those over 85 (Irvine et al., 2008). We believe that users of trazodone aged 50 and older are therefore an ideal group in which to study the long term effects of a potentially neuroprotective agent. Potential participants with any diagnosis of dementia prior to their first trazodone prescription, as well as those with diagnosed cognitive impairment, memory symptoms and confusion prior to study entry, and those with specific sub-types of dementia syndrome (Parkinson’s disease, Huntingdon’s disease, Pick’s disease, alcohol-induced dementia, dementia in other conditions, Human Immunodeficiency Virus (HIV), Lewy body disease, Cruetzfeldt-Jacob Disease) will be excluded.

**Selection of the comparison group**

To minimise treatment selection bias, we will match people in the trazodone medication group to people exposed to any other antidepressant drug that is currently recommended as monotherapy for depression in British prescribing guidelines (comparator group) using a matching algorithm based on propensity scores. Each trazodone user will be matched with up to 5 non-trazodone antidepressant users based on the propensity score using the greedy matching algorithm.

Trazodone prescribing has evolved during >15 years according to changes in treatment guidance. To be considered for inclusion, only patients present in the database from 2000 onwards and with at least 6 months follow up prior to prescribing events that trigger eligibility will be included. Follow up for the unexposed will begin at the first prescribing event that makes them eligible for inclusion.

Study variables

*Define study variables as appropriate. It is optional to append lists of drugs and diagnostic terms from THIN dictionaries (Read & Drug codes), which will be used in patient selection and analysis.*

Exposure will be determined by prescribing records, using code lists for individual antidepressant agents (BNF chapter: 4.3). The index date for each patient will be their first prescribing event that qualifies them for study entry (e.g. first exposure to an antidepressant)

The outcome for this study is the first recording of a diagnosis of dementia after the index date, as identified from clinical and referral records, using the Read code list in Appendix 1.

Analysis

*Describe techniques for analysis, including statistical methods and power calculations. Number of patients in each study group should also be stated.*

We will measure an incidence rate ratio for the association between trazodone use and incident AD using stratified Cox regression, comparing patients exposed to trazodone with patients exposed to alternative treatments. The primary analysis will follow all patients from their index date until the earliest of an dementia record, transfer out/death, or last data collection extract from practice, and their exposure will remain as defined at the index date. Patients will be censored in the event of drug switching. Sensitivity analyses will be as follows:

- Sensitivity analysis defining dementia as a Readcode indicating Alzheimer’s Disease (AD) plus at least 2 prescriptions for an cholinesterase inhibitors, to reduce the likelihood of outcome misclassification. Although cases with little follow up beyond their broad diagnosis of dementia will be excluded, this approach will only reduce study power, but will reduce misclassification bias.
- A sensitivity analysis censoring follow up at end of trazodone therapy, defined as last prescription plus 90 days, followed by at least 180 days trazodone-free follow up time.
- We have chosen to measure the incidence of dementia from the start of therapy with trazodone as the pre-clinical work to date suggests a fast onset of any protective effect (Halliday, 2017). Whether this effect varies with exposure length will be assessed through a stratified analysis on length of follow up after starting trazodone therapy. The date of earliest recorded diagnosis will remain the date of the outcome.
- A sensitivity analysis removing any events recorded within 1 month of the start of follow-up.
- A sensitivity analysis with mirtazapine only as the comparator group.
- A sensitivity analysis looking into a potential dose effect (stratifying patients by average dosage received).

All data management and analyses will be performed using Stata 12 (StataCorp, Texas) and SAS 9.4 (SAS Institute Inc, Cary, North Carolina).

Confounding variables

*As appropriate, describe methods for controlling the effects of confounding variables.*

Covariates to be included in the calculations of the propensity scores will be:

- Age (in years)
- Gender (male, female)
- Practice
- Depression diagnosis (yes, no)
- Smoking status (never, current, ex)
- Alcohol consumption (healthy use, moderate use, heavy/dependent use)
- Body mass index (normal weight, overweight, obese)
- Number of GP visits in the 12 months prior to the index date
- Other psychiatric disorders including: anxiety or sleep disorders, substance abuse, psychotic disorder, ADHD and personality disorder.
- Cardiovascular comorbidity (arrhythmias, heart failure, myocardial infarction, other acute ischemic heart disease, other chronic ischemic heart disease, hypertension, cerebrovascular disease, and diabetes mellitus)
- Other medications (antipsychotics, anti-anxiety medications and drugs listed under BNF Chapter 2 [Cardiovascular system], such as statins)
- Ethnicity
- Area level social deprivation

Validation (if this is a validation study)

Not applicable

Limitations

*Describe any likely and significant limitations to the study design, which may reduce the validity of findings. It may be beneficial to discuss any bias that may arise from the study.*

Diagnoses of dementia in primary care, including Alzheimer’s Disease, have a specificity of 83 % (Dunn et al, 2005) and so a small degree of misclassification amongst outcomes is expected. However, this is likely to be non-differential with respect to trazodone exposure and would be expected to bias results towards the null.

Misclassification of the exposure is also likely to occur to some degree, with patients not being completely adherent to prescribed medicines. However, we only include people with two or more consecutive prescriptions thereby including those patients who are most likely to be motivated to take their medication, and substantial misclassification is not anticipated. Again, misclassification is unlikely to be differential with respect to dementia status and so a dilution of any effect towards the null would be expected.

Missing data: We expect some missing data with regard to smoking status, alcohol use etc. We will run Propensity Scores (PS) on 1) complete cases and 2) he whole sample separately and use the following calibration technique to account for missing data (suggested by Til Sturmer et al: <https://www.ncbi.nlm.nih.gov/pubmed/17395595>).

Reference list

1. Protein Aggregation in the Brain: The Molecular Basis for Alzheimer’s and Parkinson’s Diseases. [GB Irvine](https://www.ncbi.nlm.nih.gov/pubmed/?term=Irvine%20GB%5BAuthor%5D&cauthor=true&cauthor_uid=18368143), [OM El-Agnaf](https://www.ncbi.nlm.nih.gov/pubmed/?term=El-Agnaf%20OM%5BAuthor%5D&cauthor=true&cauthor_uid=18368143), [GM Shankar](https://www.ncbi.nlm.nih.gov/pubmed/?term=Shankar%20GM%5BAuthor%5D&cauthor=true&cauthor_uid=18368143), and [DM Walsh](https://www.ncbi.nlm.nih.gov/pubmed/?term=Walsh%20DM%5BAuthor%5D&cauthor=true&cauthor_uid=18368143). [Mol Med](https://www.ncbi.nlm.nih.gov/pmc/articles/PMC2274891/). 2008 Jul-Aug; 14(7-8): 451–464. doi:  [10.2119/2007-00100.Irvine](https://dx.doi.org/10.2119%2F2007-00100.Irvine)
2. Repurposed drugs targeting eIF2α-P-mediated translational repression prevent neurodegeneration in mice. M[. Halliday](javascript:;), [H. Radford](javascript:;), KA[M Zents](javascript:;), C.Molloy, JA Moreno, NC Verity, E. Smith, CA Ortori, DA Barrett, M Bushell, GR Mallucci . Brain 2017 April. Doi: <https://doi.org/10.1093/brain/awx074>
3. The unfolded protein response in virus infections. SW Chan. [Front Microbiol](https://www.ncbi.nlm.nih.gov/pmc/articles/PMC4179733/). 2014; 5: 518. doi:  [10.3389/fmicb.2014.00518](https://dx.doi.org/10.3389%2Ffmicb.2014.00518)
4. Efficacy and acceptability of pharmacological treatments for depressive disorders in primary care: systematic review and network meta-analysis. Linde K, L [Kriston](https://www.ncbi.nlm.nih.gov/pubmed/?term=Kriston%20L%5BAuthor%5D&cauthor=true&cauthor_uid=25583895), G [Rücker](https://www.ncbi.nlm.nih.gov/pubmed/?term=R%C3%BCcker%20G%5BAuthor%5D&cauthor=true&cauthor_uid=25583895), S [Jamil](https://www.ncbi.nlm.nih.gov/pubmed/?term=Jamil%20S%5BAuthor%5D&cauthor=true&cauthor_uid=25583895), I [Schumann](https://www.ncbi.nlm.nih.gov/pubmed/?term=Schumann%20I%5BAuthor%5D&cauthor=true&cauthor_uid=25583895), K [Meissner](https://www.ncbi.nlm.nih.gov/pubmed/?term=Meissner%20K%5BAuthor%5D&cauthor=true&cauthor_uid=25583895), K [Sigterman](https://www.ncbi.nlm.nih.gov/pubmed/?term=Sigterman%20K%5BAuthor%5D&cauthor=true&cauthor_uid=25583895), A [Schneider](https://www.ncbi.nlm.nih.gov/pubmed/?term=Schneider%20A%5BAuthor%5D&cauthor=true&cauthor_uid=25583895). [Ann Fam Med.](https://www.ncbi.nlm.nih.gov/pubmed/25583895) 2015 Jan-Feb;13(1):69-79. doi: 10.1370/afm.1687.
5. Rediscovering trazodone for the treatment of major depressive disorder. A [Fagiolini](https://www.ncbi.nlm.nih.gov/pubmed/?term=Fagiolini%20A%5BAuthor%5D&cauthor=true&cauthor_uid=23192413) , A [Comandini](https://www.ncbi.nlm.nih.gov/pubmed/?term=Comandini%20A%5BAuthor%5D&cauthor=true&cauthor_uid=23192413), M [Catena Dell'Osso](https://www.ncbi.nlm.nih.gov/pubmed/?term=Catena%20Dell%27Osso%20M%5BAuthor%5D&cauthor=true&cauthor_uid=23192413) , S [Kasper](https://www.ncbi.nlm.nih.gov/pubmed/?term=Kasper%20S%5BAuthor%5D&cauthor=true&cauthor_uid=23192413). [CNS Drugs.](https://www.ncbi.nlm.nih.gov/pubmed/23192413) 2012 Dec;26(12):1033-49. doi: 10.1007/s40263-012-0010-5.
6. Health and Social Care Information Centre. Prescriptions Dispensed in the Community: England 2005-2015. 2016. <http://content.digital.nhs.uk/catalogue/PUB20664/pres-disp-com-eng-2005-15-rep.pdf>.
7. Association between dementia and infectious disease: evidence from a case-control study. N [Dunn](https://www.ncbi.nlm.nih.gov/pubmed/?term=Dunn%20N%5BAuthor%5D&cauthor=true&cauthor_uid=15942327), M [Mullee](https://www.ncbi.nlm.nih.gov/pubmed/?term=Mullee%20M%5BAuthor%5D&cauthor=true&cauthor_uid=15942327), VH [Perry](https://www.ncbi.nlm.nih.gov/pubmed/?term=Perry%20VH%5BAuthor%5D&cauthor=true&cauthor_uid=15942327), C [Holmes](https://www.ncbi.nlm.nih.gov/pubmed/?term=Holmes%20C%5BAuthor%5D&cauthor=true&cauthor_uid=15942327). [Alzheimer Dis Assoc Disord.](https://www.ncbi.nlm.nih.gov/pubmed/15942327) 2005 Apr-Jun;19(2):91-4.([1](#_ENREF_1))

**Appendix 1 – Code list outcome of interest**

| **readcodes** | **description** |
| --- | --- |
| Eu00.00 | [X]Dementia in Alzheimer's disease |
| Eu00000 | [X]Dementia in Alzheimer's disease with early onset |
| Eu00011 | [X]Presenile dementia,Alzheimer's type |
| Eu00012 | [X]Primary degen dementia, Alzheimer's type, presenile onset |
| Eu00013 | [X]Alzheimer's disease type 2 |
| Eu00100 | [X]Dementia in Alzheimer's disease with late onset |
| Eu00111 | [X]Alzheimer's disease type 1 |
| Eu00112 | [X]Senile dementia,Alzheimer's type |
| Eu00113 | [X]Primary degen dementia of Alzheimer's type, senile onset |
| Eu00200 | [X]Dementia in Alzheimer's dis, atypical or mixed type |
| Eu00z00 | [X]Dementia in Alzheimer's disease, unspecified |
| Eu00z11 | [X]Alzheimer's dementia unspec |
| E041.00 | Dementia in conditions EC |
| Eu04100 | [X]Delirium superimposed on dementia |
| F110.00 | Alzheimer's disease |
| F110000 | Alzheimer's disease with early onset |
| F110100 | Alzheimer's disease with late onset |
| Eu02z13 | [X] Primary degenerative dementia NOS |

| Eu02z14 | [X] Senile dementia NOS |
| --- | --- |
| Eu02z00 | [X] Unspecified dementia |
| Eu02z11 | [X] Presenile dementia NOS |
| Eu02z16 | [X] Senile dementia, depressed or paranoid type |
| Eu02z16 | [X] Senile dementia, depressed or paranoid type |
| E00..00 | Senile and presenile organic psychotic conditions |
| E00..11 | Senile dementia |
| E00..12 | Senile/presenile dementia |
| E000.00 | Uncomplicated senile dementia |
| E001.00 | Presenile dementia |
| E001000 | Uncomplicated presenile dementia |
| E001100 | Presenile dementia with delirium |
| E001200 | Presenile dementia with paranoia |
| E001300 | Presenile dementia with depression |
| E001z00 | Presenile dementia NOS |
| E002.00 | Senile dementia with depressive or paranoid features |
| E002000 | Senile dementia with paranoia |
| E002100 | Senile dementia with depression |
| E002z00 | Senile dementia with depressive or paranoid features NOS |
| E003.00 | Senile dementia with delirium |
| **Read codes to be excluded (in sensitivity analyses):** | |
| E004.00 | Arteriosclerotic dementia |
| E004.11 | Multi infarct dementia |
| E004000 | Uncomplicated arteriosclerotic dementia |
| E004100 | Arteriosclerotic dementia with delirium |
| E004200 | Arteriosclerotic dementia with paranoia |
| E004300 | Arteriosclerotic dementia with depression |
| E004z00 | Arteriosclerotic dementia NOS |
| E00y.00 | Other senile and presenile organic psychoses |
| E00y.11 | Presbyophrenic psychosis |
| E00z.00 | Senile or presenile psychoses NOS |
| Eu02.00 | [X]Dementia in other diseases classified elsewhere |
| Eu02000 | [X]Dementia in Pick's disease |
| Eu02100 | [X]Dementia in Creutzfeldt-Jakob disease |
| Eu02200 | [X]Dementia in Huntington's disease |
| Eu02300 | [X]Dementia in Parkinson's disease |
| Eu02400 | [X]Dementia in human immunodef virus [HIV] disease |
| Eu02500 | [X]Lewy body dementia |
| Eu02y00 | [X]Dementia in other specified diseases classif elsewhere |
| Eu02z12 | [X] Presenile psychosis NOS |
| Eu02z15 | [X] Senile psychosis NOS |
| Eu01.00 | [X]Vascular dementia |
| Eu01.11 | [X]Arteriosclerotic dementia |
| Eu01000 | [X]Vascular dementia of acute onset |
| Eu01100 | [X]Multi-infarct dementia |
| Eu01111 | [X]Predominantly cortical dementia |
| Eu01200 | [X]Subcortical vascular dementia |
| Eu01300 | [X]Mixed cortical and subcortical vascular dementia |
| Eu01y00 | [X]Other vascular dementia |
| Eu01z00 | [X]Vascular dementia, unspecified |
| E02y100 | Drug-induced dementia |
| E012.00 | Other alcoholic dementia |
| E012.11 | Alcoholic dementia NOS |
| E012000 | Chronic alcoholic brain syndrome |
| F111.00 | Pick's disease |
| F112.00 | Senile degeneration of brain |
| F116.00 | Lewy body disease |
