# Supplementary material for: Trazodone use and risk of dementia: A population-based cohort study
Source: PLoS Med. 2019 Feb 5;16(2):e1002728. doi: 10.1371/journal.pmed.1002728 (PMC6363148; doi:10.1371/journal.pmed.1002728)
Supplement: S1 Table — (DOCX) [file pmed.1002728.s003.docx]

**Supplemental Table 1. Drug codes for identification of antidepressant agents in The Health Improvement Network (THIN)**

| **Antidepressants** | **Drug codes** |
| --- | --- |
| Agomelatine | 82861998, 82862998 |
| Amitriptyline | 70290979, 81024979, 99472998, 99824992, 94703998, 94077990, 96328979, 97223998, 99861990, 99863990, 99864990, 99866990, 99868990, 99869990, 99870990, 99871990, 81085998, 47944978, 81084998, 92808996, 98067988, 98128998, 96924998, 94704998, 99826992, 94704997, 98343998, 94703997, 95574997, 99017998, 96925998, 94076990, 94771990, 96323979, 97223997, 99861989, 99862990, 99863989, 99864989, 99865990, 99866989, 99867989, 99868989, 99869988, 99870989, 99871989, 92808997, 96891992, 98067990, 99825992, 96925997, 94075990, 97223996, 99863988, 99864988, 99866988, 99868988, 99869989, 99870988, 99871988, 92808998, 98067989, 94067992, 96925996, 83620998, 99017997, 98129998, 98130998, 98150998, 98138998, 98130997, 98150997, 98138997, 98130996, 98150996, 98129997 |
| Amoxapine | 94005996, 94009996, 94004998, 94008998, 99791992, 94005998, 94009998, 92478998, 94005997, 94009997 |
| Bolvidon | 96987992 |
| Butriptyline | 94688998, 98134998, 94688997 |
| Citalopram | 69605979, 69606979, 69604979, 87251998, 91380997, 91395997, 93948990, 93994990, 94895990, 95271990, 95335990, 95421990, 95633990, 95668990, 95705990, 95995979, 91380998, 91395998, 93947990, 93996990, 94603990, 94894990, 94937990, 95270990, 95334990, 95420990, 95632990, 95667990, 95704990, 91380996, 91395996, 93946990, 94880990, 94893990, 94936990, 95269990, 95333990, 95418990, 95631990, 95666990, 95703990, 95979979, 95984979, 92172998, 92174998 |
| Clomipramine | 80548979, 96640998, 97548990, 98144998, 98340990, 99297990, 97167992, 96640997, 96901989, 97548989, 97773989, 98144997, 98340989, 99297989, 96639998, 98143998, 80550979, 93358992, 96640996, 96901988, 97548988, 98144996, 98340988, 99297988, 83878998, 96638998, 93360992, 96637998, 99794992, 98142998 |
| Desipramine | 96442998, 98146998 |
| Dosulepin | 82640998, 98783990, 88906998, 94801990, 96158990, 96282979, 96311998, 96467990, 96868990, 96964990, 97722998, 97762990, 97818998, 98126998, 98351989, 98563989, 99614990, 98327997, 80274979, 96311996, 98078990, 88906997, 94800990, 95247990, 95248990, 96311997, 96868989, 97722997, 97762989, 97818997, 98126997, 98351990, 98563990, 99614989, 80278979, 98078989, 98327998 |
| Dothiepin | 94940992, 94941992, 94146992, 94944992, 94943992, 94942992, 94452992, 94145992 |
| Doxepin | 80210979, 96308998, 98124998, 85172998, 96308997, 98124997, 85171998, 96308996, 98124996, 96307998, 98123998 |
| Duloxetine | 39667978, 86997998, 86999998, 89023979, 37600978, 51109978, 86996998, 86998998 |
| Escitalopram | 88285998, 89381979, 89383979, 91671998, 85970998, 85971998, 98088998, 98561998, 82790998, 82791998, 87662998, 87663998 |
| Fluoxetine | 30932978, 82367998, 80064979, 80062979, 30041978, 29604978, 30444978, 90159998, 90814998, 93066990, 93905990, 94447998, 94490998, 95388990, 96161979, 96162979, 96168979, 96272990, 96281990, 96606990, 96643990, 96644990, 96647990, 96651990, 96654990, 96659990, 96674990, 96709990, 96729990, 99592998, 75904978, 75905978, 76398978, 84403998, 90766998, 91923990, 91928990, 94447997, 94490997, 95426990, 95813990, 95820990, 96155979, 84436998, 94447996, 94490996, 95610990, 96143979 |
| Flupentixol | 96504997, 99634997, 96504998, 99634998 |
| Fluphenazine | 97632998, 96499998, 97634998 |
| Fluvoxamine | 96345989, 96492997, 96493997, 96810989, 96093990, 96492998, 96493998 |

**Supplemental Table 1. Drug codes for identification of antidepressant agents in The Health Improvement Network (THIN) (continued)**

| **Antidepressants** | **Drug codes** |
| --- | --- |
| Imipramine | 96687992, 97112998, 99554990, 99555990, 95155992, 96265979, 97112997, 98140997, 98149990, 99554989, 99555989, 99556989, 96130998, 62948979, 82432998, 95156992, 95154992, 97593992, 97091998, 98140998, 98140996 |
| Iprindole | 96108998, 96109998, 96109997 |
| Iproniazid | 96107998, 99448998 |
| Isocarboxazid | 96105998, 97169990, 99450998 |
| L-tryptophan | 99937992 |
| Lofepramine | 67063979, 95999998, 96793990, 96855990, 96963990, 97142990, 97192990, 97743990, 97861990 |
| Lofepramine | 98132998, 89205998, 95999997, 98077990 |
| Maprotiline | 95928998, 95928997, 95928996, 95927998, 97704992, 98148998, 98148997, 98148996, 98147998 |
| Merital | 94498992, 94234992, 95262992 |
| Mianserin | 95809998, 95809997, 99494989, 95809996, 99338998, 99882998, 99338997, 99882997, 99338996, 99882996 |
| Mirtazapine | 58747979, 87685998, 87687998, 90119979, 90125979, 92454990, 92906990, 92981990, 92988990, 92994990, 93180990, 86982998, 92814990, 94037990, 94250990, 94401990, 87430998, 94870990, 87945998, 87946998, 90094979, 90097979, 90105979, 92980990, 88715998, 88717998, 94126990, 94611990, 94773990, 94797990, 94847990, 95949979, 87684998, 58745979, 86981998, 87686998, 92813990, 92903990, 92979990, 92986990, 92992990, 93178990, 94035990, 94400990 |
| Moclobemide | 93749998, 93759998, 96061990, 96199979, 93749997, 93759997 |
| Nefazodone | 91361998, 91362998, 91361997, 91362997, 91362996, 91361996 |
| Nomifensine | 97807992, 96365992 |
| Nortriptyline | 64091979, 94249992, 94630998, 95695998, 92015990, 95696998, 96248979, 98152998, 95695996, 95695997, 92014990, 95696997, 96244979, 98152997, 94630997, 98154998, 98154996, 98154997 |
| Paroxetine | 66539979, 29586978, 54494979, 54495979, 84807998, 85382998, 66541979, 93489996, 93490996, 96068979, 96070979, 93489998, 93490998, 95051990, 95332990, 95350990, 95578990, 96087990, 96098979, 93487990, 93489997, 93490997, 94852990, 95007990, 95028990, 96082979 |
| Perphenazine | 95574998 |
| Phenelzine | 95560998, 99377998 |
| Protriptyline | 95372997, 95372998, 90000979, 96416992, 97505998, 97507998 |
| Reboxetine | 88836998, 88838998 |
| Sertraline | 79261979, 66189979, 66187979, 66185979, 66183979, 52706979, 60187979, 92729990, 93173997, 93174997, 93732990, 93752990, 93842990, 96114979, 96118979, 60188979, 92728990, 93173998, 93174998, 93694990, 93733990, 93749990, 93753990, 93843990, 96136979, 86159998 |
| Sinequan | 98027992 |
| Tofranil | 98183992 |
| Tranylcypromi | 99280998, 95144998, 95665990, 99281998, 95143998 |
| Trazodone | 65273979, 65269979, 65265979, 65263979, 65261979, 65255979, 65249979, 95142997, 96295989, 96422989, 96443989, 96726989, 98486997, 95141997, 95142996, 95527990, 96295988, 96422988, 96443988, 98486996, 95142998, 96295990, 96422990, 96443990, 96726990, 98486998, 65251979, 65253979, 83781998, 91934990, 95141998, 98312998, 98312997 |
| Trifluoperazi | 94626998 |

**Supplemental Table 1. Drug codes for identification of antidepressant agents in The Health Improvement Network (THIN) (continued)**

| **Antidepressants** | **Drug codes** |
| --- | --- |
| Trimipramine | 65015979, 65013979, 93841990, 95107998, 98136998, 93840990, 95107997, 98136997, 93839990, 95107996, 98136996, 98212992 |
| Tryptophan | 52984979, 52985979, 94512992, 94663992, 95099998, 95352992, 98257998, 99294998, 99316998, 95098997 |
| Venlafaxine | 64976979, 30261978, 79303978, 79304978, 64642979, 64640979, 64638979, 52700979, 81749998, 81929998, 82190998, 82874998, 83074998, 83114998, 83145998, 83149998, 83204998, 83209998, 83217998, 83264998, 88755997, 88776997, 96022979, 96023979, 96024979, 96029979, 52165979, 80024978, 82962998, 83157998, 83159998, 39137978, 39138978, 82959998, 82961998, 81505998, 81506998, 83163998, 92597990, 96059979, 96065979, 98336998, 99896998, 86431998, 98336996, 99896996, 81750998, 81930998, 82191998, 82540998, 82875998, 83075998, 83115998, 83146998, 83150998, 83205998, 83210998, 83218998, 83265998, 88755998, 88776998, 96033979, 96034979, 96036979, 96041979, 52164979, 80023978, 82963998, 83158998, 83160998, 83162998, 92596990, 96052979, 96054979, 98336997, 99896997 |
| Viloxazine | 98959998, 95624998 |
| Vortioxetine | 45795978, 45796978, 45793978, 45794978, 45791978, 45792978 |
| Zimelidine | 98327992 |
