# Supplementary material for: Trazodone use and risk of dementia: A population-based cohort study
Source: PLoS Med. 2019 Feb 5;16(2):e1002728. doi: 10.1371/journal.pmed.1002728 (PMC6363148; doi:10.1371/journal.pmed.1002728)
Supplement: S2 Table — (DOCX) [file pmed.1002728.s004.docx]

**Supplemental Table 2. Read codes for identification of dementia in The Health Improvement Network (THIN)**

| **Read codes** | **Descriptions** |
| --- | --- |
| **Dementia (primary outcome)** | |
| E00..00 | Senile and presenile organic psychotic conditions |
| E00..11 | Senile dementia |
| E00..12 | Senile/presenile dementia |
| E000.00 | Uncomplicated senile dementia |
| E001.00 | Presenile dementia |
| E001000 | Uncomplicated presenile dementia |
| E001100 | Presenile dementia with delirium |
| E001200 | Presenile dementia with paranoia |
| E001300 | Presenile dementia with depression |
| E001z00 | Presenile dementia NOS |
| E002.00 | Senile dementia with depressive or paranoid features |
| E002000 | Senile dementia with paranoia |
| E002100 | Senile dementia with depression |
| E002z00 | Senile dementia with depressive or paranoid features NOS |
| E003.00 | Senile dementia with delirium |
| E004.00 | Arteriosclerotic dementia |
| E004.11 | Multi infarct dementia |
| E004000 | Uncomplicated arteriosclerotic dementia |
| E004100 | Arteriosclerotic dementia with delirium |
| E004200 | Arteriosclerotic dementia with paranoia |
| E004300 | Arteriosclerotic dementia with depression |
| E004z00 | Arteriosclerotic dementia NOS |
| E041.00 | Dementia in conditions EC |
| Eu00.00 | [X]Dementia in Alzheimer's disease |
| Eu00000 | [X]Dementia in Alzheimer's disease with early onset |
| Eu00011 | [X]Presenile dementia,Alzheimer's type |
| Eu00012 | [X]Primary degen dementia, Alzheimer's type, presenile onset |
| Eu00013 | [X]Alzheimer's disease type 2 |
| Eu00100 | [X]Dementia in Alzheimer's disease with late onset |
| Eu00111 | [X]Alzheimer's disease type 1 |
| Eu00112 | [X]Senile dementia,Alzheimer's type |
| Eu00113 | [X]Primary degen dementia of Alzheimer's type, senile onset |
| Eu00200 | [X]Dementia in Alzheimer's dis, atypical or mixed type |
| Eu00z00 | [X]Dementia in Alzheimer's disease, unspecified |
| Eu00z11 | [X]Alzheimer's dementia unspec |
| Eu01.00 | [X]Vascular dementia |
| Eu01.11 | [X]Arteriosclerotic dementia |
| Eu01000 | [X]Vascular dementia of acute onset |
| Eu01100 | [X]Multi-infarct dementia |
| Eu01111 | [X]Predominantly cortical dementia |
| Eu01200 | [X]Subcortical vascular dementia |
| Eu01300 | [X]Mixed cortical and subcortical vascular dementia |
| Eu01y00 | [X]Other vascular dementia |

**Supplemental Table 2. Read codes for identification of dementia in The Health Improvement Network (THIN) (continued)**

| **Read codes** | **Descriptions** |
| --- | --- |
| **Dementia (primary outcome) (continued)** | |
| Eu01z00 | [X]Vascular dementia, unspecified |
| Eu02z00 | [X] Unspecified dementia |
| Eu02z11 | [X] Presenile dementia NOS |
| Eu02z13 | [X] Primary degenerative dementia NOS |
| Eu02z14 | [X] Senile dementia NOS |
| Eu02z16 | [X] Senile dementia, depressed or paranoid type |
| Eu04100 | [X]Delirium superimposed on dementia |
| F110.00 | Alzheimer's disease |
| F110000 | Alzheimer's disease with early onset |
| F110100 | Alzheimer's disease with late onset |
| Fyu3000 | [X]Other Alzheimer's disease |
| 66h..00 | Dementia monitoring |
| 6AB..00 | Dementia annual review |
| 9Ou..00 | Dementia monitoring administration |
| 9Ou1.00 | Dementia monitoring first letter |
| 9Ou2.00 | Dementia monitoring second letter |
| 9Ou3.00 | Dementia monitoring third letter |
| 9Ou4.00 | Dementia monitoring verbal invite |
| 9Ou5.00 | Dementia monitoring telephone invite |
| 9hD..00 | Exception reporting: dementia quality indicators |
| 9hD0.00 | Excepted from dementia quality indicators: Patient unsuitabl |
| 9hD1.00 | Excepted from dementia quality indicators: Informed dissent |
| ZS7C500 | Language disorder of dementia |
| 1461.00 | H/O: dementia |
|  |  |
| **Alzheimer’s disease dementia** (strict outcome definition in sensitivity analysis) | |
| Eu00.00 | [X]Dementia in Alzheimer's disease |
| Eu00000 | [X]Dementia in Alzheimer's disease with early onset |
| Eu00011 | [X]Presenile dementia,Alzheimer's type |
| Eu00012 | [X]Primary degen dementia, Alzheimer's type, presenile onset |
| Eu00013 | [X]Alzheimer's disease type 2 |
| Eu00100 | [X]Dementia in Alzheimer's disease with late onset |
| Eu00111 | [X]Alzheimer's disease type 1 |
| Eu00112 | [X]Senile dementia,Alzheimer's type |
| Eu00113 | [X]Primary degen dementia of Alzheimer's type, senile onset |
| Eu00200 | [X]Dementia in Alzheimer's dis, atypical or mixed type |
| Eu00z00 | [X]Dementia in Alzheimer's disease, unspecified |
| Eu00z11 | [X]Alzheimer's dementia unspec |
| F110.00 | Alzheimer's disease |
| F110000 | Alzheimer's disease with early onset |
| F110100 | Alzheimer's disease with late onset |
| Fyu3000 | [X]Other Alzheimer's disease |

**Supplemental Table 2. Read codes for identification of dementia in The Health Improvement Network (THIN) (continued)**

| **Read codes** | **Descriptions** |
| --- | --- |
| **Dementia with identifiable causes other than Alzheimer’s disease and vascular dementia** (patients with these diagnosis codes were censored at the date of the diagnosis and did not count towards the total number of outcome events) | |
|  | |
| E012.00 | Other alcoholic dementia |
| E012.11 | Alcoholic dementia NOS |
| Eu02.00 | [X]Dementia in other diseases classified elsewhere |
| Eu02000 | [X]Dementia in Pick's disease |
| Eu02100 | [X]Dementia in Creutzfeldt-Jakob disease |
| Eu02200 | [X]Dementia in Huntington's disease |
| Eu02300 | [X]Dementia in Parkinson's disease |
| Eu02400 | [X]Dementia in human immunodef virus [HIV] disease |
| Eu02y00 | [X]Dementia in other specified diseases classif elsewhere |
| Eu10711 | [X]Alcoholic dementia NOS |
| F111.00 | Pick's disease |
|  |  |
