# Supplementary material for: Trazodone use and risk of dementia: A population-based cohort study
Source: PLoS Med. 2019 Feb 5;16(2):e1002728. doi: 10.1371/journal.pmed.1002728 (PMC6363148; doi:10.1371/journal.pmed.1002728)
Supplement: S3 Table — (DOCX) [file pmed.1002728.s005.docx]

**Supplemental Table 3. Baseline characteristics of individuals with complete and incomplete data**

|  | Complete cases  (N=239,268) | Incomplete cases  (either with missing smoking status, alcohol status, BMI, or Townsend score)  (N=185,728) | Standardised difference |
| --- | --- | --- | --- |
| Age, mean ± standard deviation | 64.3 ± 10.5 | 67.5 ± 12.2 | 0.28 |
| Sex (Female) | 143,914 (60.1) | 109,610 (59.0) | 0.02 |
| Cerebrovascular disease | 15,915 (6.7) | 17,728 (9.5) | 0.11 |
| Diabetes | 29,989 (12.5) | 19,173 (10.3) | 0.07 |
| Arrhythmias | 15,225 (6.4) | 13,795 (7.4) | 0.04 |
| Myocardial infarction | 43,231 (18.1) | 32,485 (17.5) | 0.02 |
| Hypertension | 97,689 (40.8) | 64,279 (34.6) | 0.13 |
| Heart failure | 6,122 (2.6) | 9,101 (4.9) | 0.12 |
| Personality disorder | 1,299 (0.5) | 1,229 (0.7) | 0.02 |
| ADHD | 29 (0.01) | 14 (0.01) | 0.005 |
| Psychotic disorder | 2,266 (0.9) | 2,497 (1.3) | 0.04 |
| Substance abuse | 6,919 (2.9) | 5,611 (3.0) | 0.01 |
| Sleep disorder | 5,987 (2.5) | 3,190 (1.7) | 0.05 |
| Anxiety | 45,705 (19.1) | 29,964 (16.1) | 0.08 |
| Depression | 86,829 (36.3) | 63,581 (34.2) | 0.04 |
| Antipsychotic drug use |  |  |  |
| current user | 4,254 (1.8) | 6,674 (3.6) | 0.12 |
| past user | 2,024 (0.8) | 2,009 (1.1) |  |
| non-user | 232,990 (97.4) | 1,77,045 (95.3) |  |

**Supplemental Table 3. Baseline characteristics of individuals with complete and incomplete data (continued)**

|  | Complete cases  (N=239,268) | Incomplete cases  (either with missing smoking status, alcohol status, BMI, or Townsend score)  (N=185,728) | Standardised difference |
| --- | --- | --- | --- |
| Cardiovascular drug use |  |  |  |
| Current user | 146,438 (61.2) | 110,491 (59.5) | 0.06 |
| Past user | 12,962 (5.4) | 8,373 (4.5) |  |
| Non-user | 79,868 (33.4) | 66,864 (36.0) |  |
| Anxiolytics drug use |  |  |  |
| Current user | 53,455 (22.3) | 45,686 (24.6) | 0.12 |
| Past user | 31,526 (13.2) | 17,881 (9.6) |  |
| Non-user | 154,287 (64.5) | 122,161 (65.8) |  |
| Number of general practice visits in 12 months prior to the index date, mean ± standard deviation | 29.5 ± 20.3 | 26.7 ± 20.7 | 0.14 |

Abbreviations: ADHD, Attention Deficit Hyperactivity Disorder; BMI, body mass index.

Values are expressed as count (percentage) unless otherwise specified. Standardised difference is the absolute difference in means (for continuous variables) or proportions (for categorical variables) between trazodone users and other antidepressants users divided by the pooled standard deviation.
