# Supplementary material for: Trazodone use and risk of dementia: A population-based cohort study
Source: PLoS Med. 2019 Feb 5;16(2):e1002728. doi: 10.1371/journal.pmed.1002728 (PMC6363148; doi:10.1371/journal.pmed.1002728)
Supplement: S4 Table — (DOCX) [file pmed.1002728.s006.docx]

**Supplemental Table 4. Results of the complete case analyses (trazodone vs other antidepressants)**

|  | **Crude estimates** | | **Adjusted estimates** | |
| --- | --- | --- | --- | --- |
| Type of analysis | Hazard Ratio (95% CI) | P Value | Hazard Ratio (95% CI) | P Value |
| Primary analysis | 1.51 (1.26-1.81) | <.001 | 1.64 (1.29-2.08) | <.001 |
| Secondary analyses |  |  |  |  |
| Alzheimer’s dementia only | 0.76 (0.43-1.34) | 0.34 | 0.62 (0.31-1.23) | 0.17 |
| Follow-up time censored after antidepressant use | 2.50 (1.98-3.16) | <.001 | 2.97 (2.10-4.21) | <.001 |
| Trazodone vs mirtazapine | 0.82 (0.67-1.01) | 0.06 | 1.29 (0.79-2.11) | 0.31 |
| Time-updated follow-up periods: |  |  |  |  |
| Current | 2.45 (1.95-3.08) | <.001 | 2.85 (2.08-3.91) | <.001 |
| Current <2 years | 3.28 (2.40-4.48) | <.001 | 3.41 (2.26-5.14) | <.001 |
| Current 2-3 years | 3.19 (1.71-5.95) | <.001 | 3.60 (1.45-8.95) | 0.01 |
| Current >3 years | 1.65 (1.11-2.44) | 0.01 | 1.53 (0.78-3.00) | 0.22 |
| Past | 1.09 (0.80-1.47) | 0.58 | 1.28 (0.81-2.02) | 0.29 |
| Exclusion of recent dementia: |  |  |  |  |
| Dementia diagnosis <31 days of follow-up | 1.47 (1.22-1.77) | <.001 | 1.59 (1.25-2.03) | <.001 |
| Dementia diagnosis <180 days of follow-up | 1.36 (1.12-1.65) | 0.002 | 1.40 (1.08-1.82) | 0.01 |
| Dementia diagnosis <365 days of follow-up | 1.25 (1.01-1.54) | 0.04 | 1.31 (0.99-1.73) | 0.06 |

Abbreviation: CI: Confidence interval.

Crude estimates were obtained before propensity score matching; adjusted estimates were obtained after propensity score matching.
