# Supplementary material for: Trazodone use and risk of dementia: A population-based cohort study
Source: PLoS Med. 2019 Feb 5;16(2):e1002728. doi: 10.1371/journal.pmed.1002728 (PMC6363148; doi:10.1371/journal.pmed.1002728)

**Supplemental Figure 1. Distribution of the observed and imputed values**
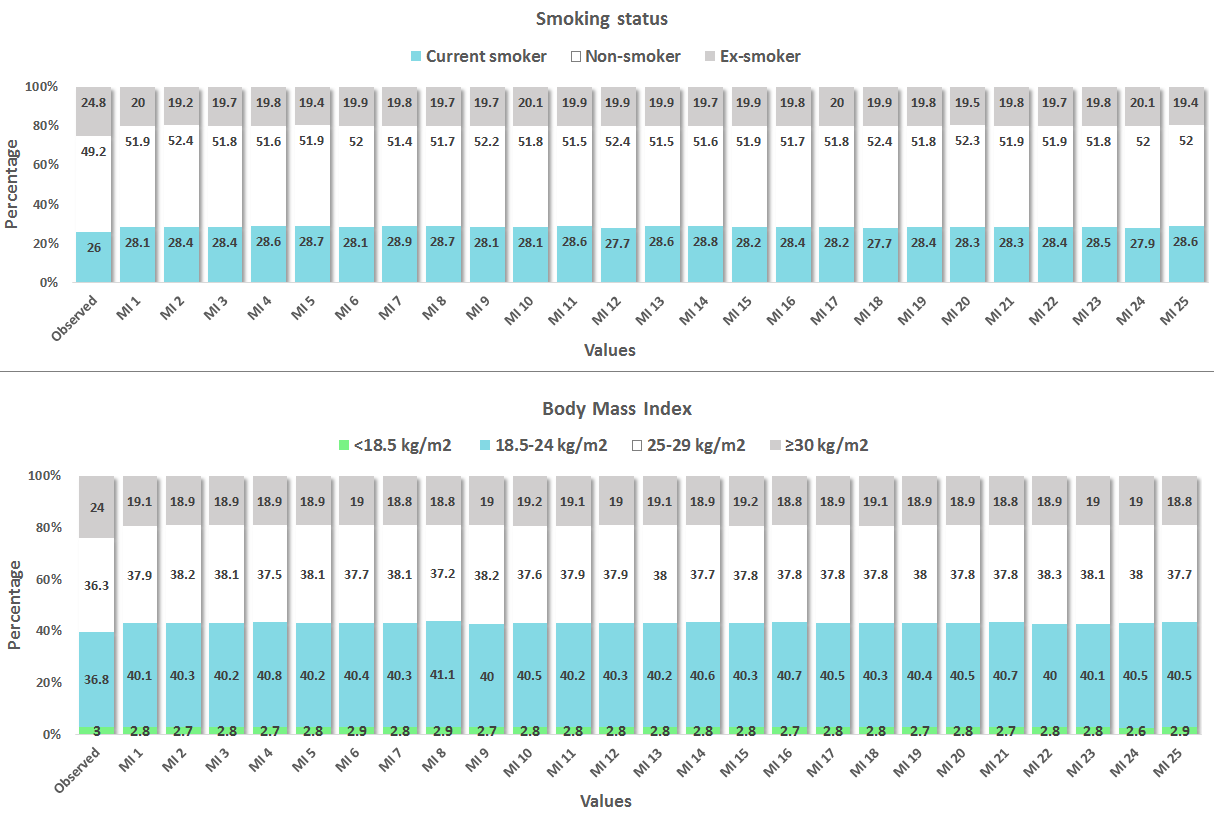


**Supplemental Figure 1. Distribution of the observed and imputed values (MI1-MI25 denote the 1^st^ to 25^th^ imputed datasets generated after multiple imputation) (continued)**


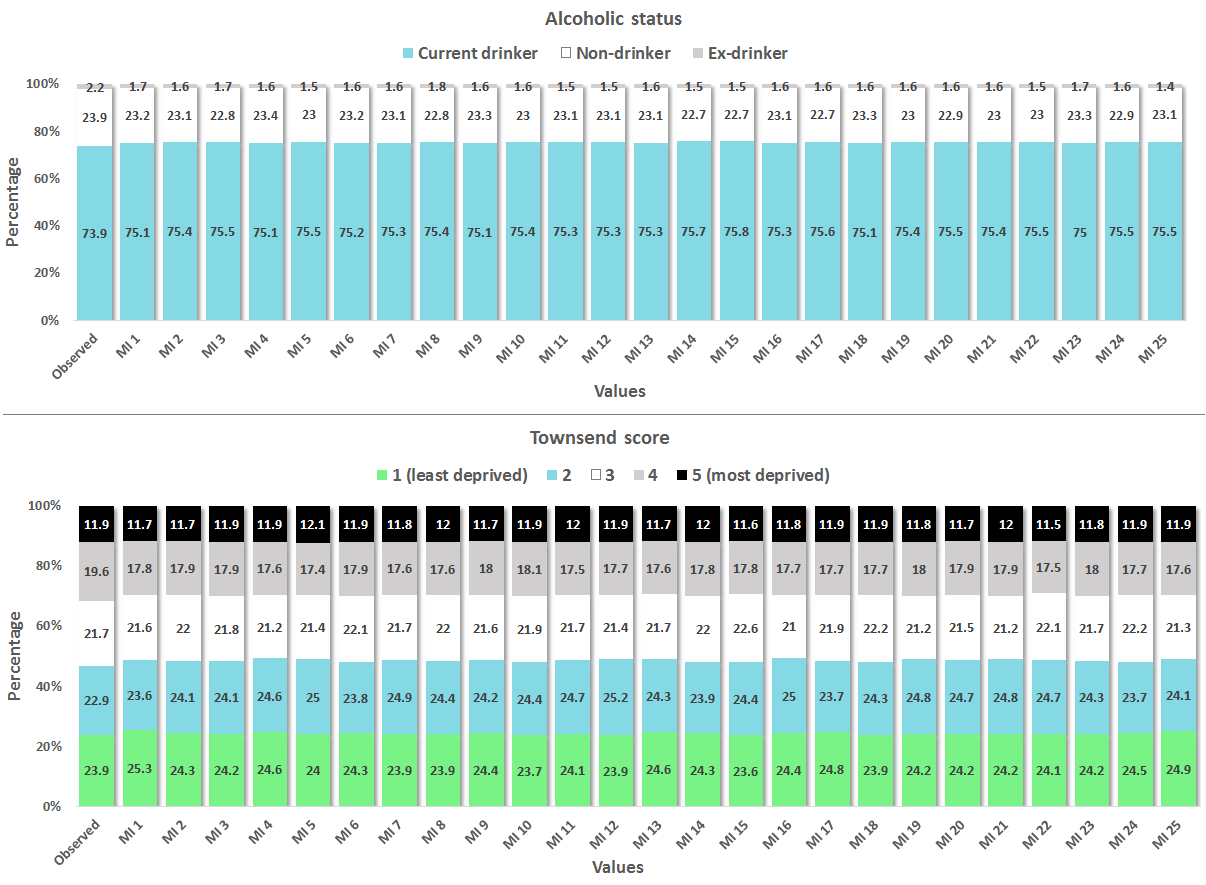

Supplement: S1 Fig — (DOCX) [file pmed.1002728.s007.docx]
